# Identification of G-quadruplex forming sequences in three manatee papillomaviruses

**DOI:** 10.1371/journal.pone.0195625

**Published:** 2018-04-09

**Authors:** Maryam Zahin, William L. Dean, Shin-je Ghim, Joongho Joh, Robert D. Gray, Sujita Khanal, Gregory D. Bossart, Antonio A. Mignucci-Giannoni, Eric C. Rouchka, Alfred B. Jenson, John O. Trent, Jonathan B. Chaires, Julia H. Chariker

**Affiliations:** 1 James Graham Brown Cancer Center, University of Louisville, Louisville, Kentucky, United States of America; 2 Department of Medicine, University of Louisville, Louisville, Kentucky, United States of America; 3 Department of Biochemistry and Molecular Genetics, University of Louisville, Louisville, Kentucky, United States of America; 4 Georgia Aquarium, Atlanta, Georgia, United States of America; 5 Division of Comparative Pathology, Department of Pathology, Miller School of Medicine, University of Miami, Miami, Florida, United States of America; 6 Puerto Rico Manatee Conservation Center, Inter American University of Puerto Rico, Bayamon, Puerto Rico; 7 Department of Computer Engineering and Computer Science, University of Louisville, Duthie Center for Engineering, Louisville, Kentucky, United States of America; 8 KBRIN Bioinformatics Core, University of Louisville, Louisville, Kentucky, United States of America; 9 Department of Psychological and Brain Sciences, University of Louisville, Louisville, Kentucky, United States of America; International Centre for Genetic Engineering and Biotechnology, ITALY

## Abstract

The Florida manatee (*Trichechus manatus latirotris*) is a threatened aquatic mammal in United States coastal waters. Over the past decade, the appearance of papillomavirus-induced lesions and viral papillomatosis in manatees has been a concern for those involved in the management and rehabilitation of this species. To date, three manatee papillomaviruses (TmPVs) have been identified in Florida manatees, one forming cutaneous lesions (TmPV1) and two forming genital lesions (TmPV3 and TmPV4). We identified DNA sequences with the potential to form G-quadruplex structures (G4) across the three genomes. G4 were located on both DNA strands and across coding and non-coding regions on all TmPVs, offering multiple targets for viral control. Although G4 have been identified in several viral genomes, including human PVs, most research has focused on canonical structures comprised of three G-tetrads. In contrast, the vast majority of sequences we identified would allow the formation of non-canonical structures with only two G-tetrads. Our biophysical analysis confirmed the formation of G4 with parallel topology in three such sequences from the E2 region. Two of the structures appear comprised of multiple stacked two G-tetrad structures, perhaps serving to increase structural stability. Computational analysis demonstrated enrichment of G4 sequences on all TmPVs on the reverse strand in the E2/E4 region and on both strands in the L2 region. Several G4 sequences occurred at similar regional locations on all PVs, most notably on the reverse strand in the E2 region. In other cases, G4 were identified at similar regional locations only on PVs forming genital lesions. On all TmPVs, G4 sequences were located in the non-coding region near putative E2 binding sites. Together, these findings suggest that G4 are possible regulatory elements in TmPVs.

## Introduction

**Fig 1:**
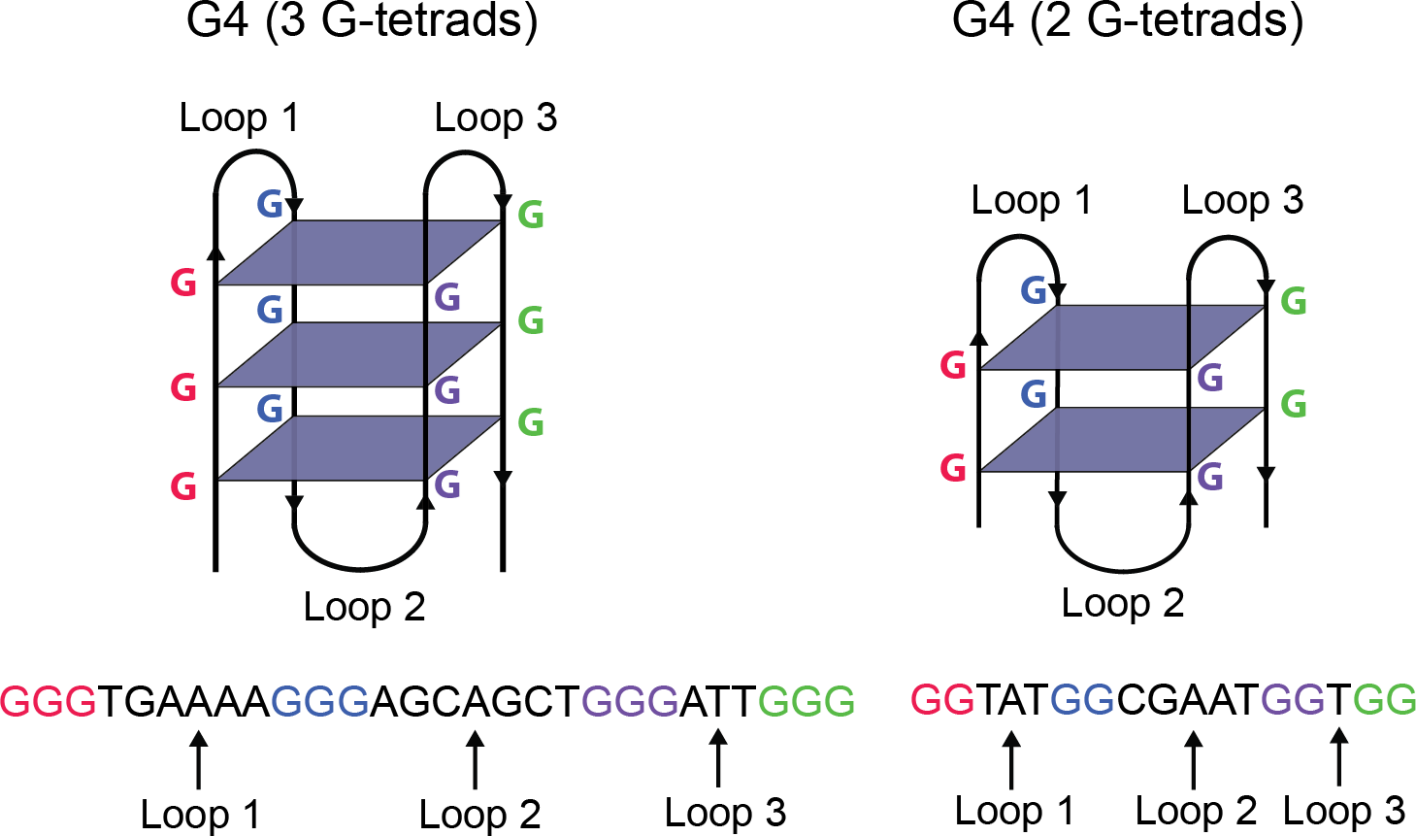
**Secondary intramolecular G4 structures (top) and corresponding DNA sequences (bottom) with varying numbers of G-tetrads.** This Figure has been modified from [87].

G-quadruplex structures (G4) are four-stranded, inter- and intramolecular structures formed from guanine-rich DNA and RNA sequences. The sequences can be identified by four tracts of two or more guanine bases that are separated by one or more nucleotides of varying composition. The sequences fold to form stacks of G-tetrads, planar structures composed of four guanine bases held together by Hoogsteen hydrogen bonds (Fig 1) [1]. The stacked G-tetrads are connected by loops which vary in size and sequence composition. The number of G-tetrads, the loop lengths, and the loop sequence composition are all known to affect the stability of the folded structure, with higher numbers of G-tetrads and shorter loop lengths tending toward greater stability [2, 3].

G4 are known to be involved in a series of key biological functions. In humans, G4 are found in telomeric repeats and serve to prevent degradation and genomic instability [4]. Their formation in this region is also known to decrease telomerase activity which is selectively expressed in a vast majority of cancers [5]. G4 located in the promoter region of genes act as transcriptional regulators [6] while those found in intronic and exonic regions play a role in alternative splicing [7, 8]. In RNA, G4 identified in 3’ and 5’ untranslated regions are known to regulate protein synthesis [9, 10].

G4 function as regulators through at least a couple of different mechanisms [1]. Their formation can inhibit transcription by blocking the activity of RNA polymerase. Alternatively, G4 can bind with other regulatory elements that either activate or repress transcription. A variety of different proteins are now known to bind with G4 in DNA and RNA [11]. In RNA, this includes proteins involved in splicing as well as protein synthesis [12].

Although much of the research on G4 is focused on humans, a regulatory role for G4 in prokaryotic cells is currently being established. In *Escherichia coli*, G4 are capable of forming and altering expression levels [13], and in *Neisseria gonorrhea*, a G4 structure plays a critical role in antigenic variation [14]. Several G4 sequences have been identified in the promotor region of the human immunodeficiency virus 1 (HIV-1) where ligand stabilization of a G4 located in the *Nef* gene reduced gene expression and repressed HIV-1 infectivity in an antiviral assay [15–21]. A G4 in the Epstein-Barr virus (EBV) altered the synthesis of EBNA1, a protein associated with processes involved in avoiding immune detection. Production of EBNA1 was reduced when the G4 was stabilized with a ligand and enhanced when destabilizing the G4 [22]. Recently, ligand stabilization of G4 in Herpes Simplex virus 1 (HSV-1) inhibited viral DNA replication [23]. G4 have also been detected in human papillomaviruses (HPV) [24], hepatitis C [25], Zika virus [26], and Ebola virus [27].

In HPV, researchers searched for canonical sequence patterns capable of forming G4 with three or more G-tetrads [24] and identified G4 in four coding and non-coding regions (NCR, L2, E1, and E4) of eight HPV types, a surprisingly small number given that there are well over a hundred HPV types [28]. Their ability to form secondary structures in the laboratory was established. However, it remains unclear how G4 affect HPV replication and transcription [12, 24].

In the current paper, we identify and characterize the first G4 sequences in papillomaviruses infecting a non-human animal, the Florida manatee. This analysis was motivated by work in our laboratory suggesting the formation of a G4 during initial sequencing of one of three manatee papillomavirus genomes. Interestingly, the sequences creating difficulty were capable of forming only non-canonical structures with two G-tetrads. In our computational analysis, we found more potential structures comprised of two G-tetrads than was found in the previous study of G4 in HPV where researchers searched only for structures with three G-tetrads. In our biophysical analysis, we found that sequences in the E2 coding region where we encountered sequencing difficulty were capable of forming G4 with a parallel topology, and in fact, they appeared to be forming multiple two G-tetrad structures stacked upon one another. In the following paragraphs, we briefly describe the ecological relevance of G4 in papillomaviruses infecting manatees and provide evidence for the biological relevance of G4 with two G-tetrads to further justify extending our search to include these structures.

The Florida manatee is an aquatic mammal living in the coastal waters of Florida that has been classified as an endangered species since 1967. Its population declined for a variety of reasons, not the least of which was that its gentle, slow-moving nature made it vulnerable to injury from boat propellers. Efforts at restoring the population have been successful to the extent that the species was downlisted to threatened status in 2016. However, in the midst of these efforts, animals undergoing rehabilitation frequently showed signs of high sensitivity to environmental stress, one sign being the development of cutaneous or mucosotropic genital papillomatous lesions. Some animals, both in captivity and in the wild, showed antibody titers indicating the presence or exposure to Trichechus manatus PV 1 (TmPV1), a virus first characterized in our laboratory [29–31]. More recently, genital lesions appeared in a single Florida manatee used as a surrogate animal for manatee rehabilitation at the Puerto Rico Manatee Conservation Center (PRMCC), and DNA sequencing, also performed in our laboratory, indicated the presence of two new PVs, *Trichechus manatus* PV 3 (TmPV3) and 4 (TmPV4) [31, 32]. These are the first known genital mucosotropic PVs in a manatee, presenting a novel sexually transmitted viral medical condition in this species should the virus spread in wild populations, where its presence is not currently known but suspected to occur naturally in the wild populations of the species throughout its range.

Similar to HPVs, manatee PV genomes are comprised of double stranded DNA, approximately 8 Kb in length that encodes a maximum of seven genes. Five genes encode non-structural or early proteins E1, E2, E4, E6 and E7, and two encode structural or late proteins L1 and L2, with all coding regions located on the forward DNA strand. A non-coding region holds the origin of replication and at least a couple of promotor sites. Much of what is known about the function of these sites comes from molecular biological research on human PVs [33]. E1 and E2 proteins form a complex that initiates viral replication at the origin, resulting in amplification of the virus. E2 also functions as a negative regulator of E6 and E7, two coding regions that stimulate cell growth and function as oncogenes in human PVs. The late proteins L1 and L2 code for the major and minor capsid proteins that enclose viral DNA, with E4 having a possible role in facilitating virion release.

During our initial sequencing of TmPV4, a glycine rich GGA repeat sequence identified in the E2 region created an obstacle to sequencing due to the formation of a secondary structure, necessitating the use of power-read sequencing analysis to complete the genome [31, 32]. We reasoned this was possibly a G4, given that a GGA repeat would be a sequence pattern capable of G4 formation. It is important to note, however, that a GGA repeat would support the formation of a G4 with only two G-tetrads. Until recently, the vast majority of research on G4 has focused on sequence patterns capable of forming structures with three or more G-tetrads and loop lengths of one to seven bases. When Huppert and Balusubramanian (2005) first made a genome-wide search for G4 using computational means, they restricted their search to this pattern as a necessary constraint on a complex search task [34]. This was based on laboratory research indicating that this sequence pattern would produce structures of greater stability, but it was acknowledged that G4 could form from other sequence patterns. Nevertheless, over the years, research has largely focused on structures with three or more G-tetrads.

Evidence is slowly accumulating that structures with two G-tetrads have biological relevance. In the studies of *E*. *coli*, HIV-1, and EBV mentioned earlier, sequences capable of only two G-tetrads formed secondary structures, and in *E*. *coli* and EBV, their formation altered expression levels. Furthermore, GGA repeat sequences are known to form intramolecular parallel G4 in the laboratory [35], and in a study of secondary structures formed from triplet repeat RNAs, AGG and UGG repeats also formed stable G4 [36]. In the human c-*myb* gene, a G4 formed from a GGA repeat sequence blocked transcription [37], and may serve as a negative regulator of gene expression [38]. It is also the case that formation of these structures can have useful clinical applications as seen with two thrombin binding aptamers shown to form two G-tetrad structures. These aptamers bind to different sites on the human thrombin protein, leading to inhibition of clot formation [39, 40].

In the human genome, Qin et al (2015) find a greater number of two G-tetrad sequences than three G-tetrad sequences. Although this might be expected given the greater likelihood of shorter G-tracts occurring at random [41], their examination of several sequence patterns indicates that secondary structures do form with two G-tetrads. Structural conformation and thermal stability is dependent on loop length and composition as is seen with three G-tetrad structures. G4 with two G-tetrads, as well as other types of non-canonical structures, have also been identified using new high throughput sequencing methods, G4-seq and rG4-seq, which are the first genome- and transcriptome-wide methods for detecting formed, rather than predicted, secondary structures [42, 43]. Using G4-seq, Chambers et al. (2015) identified 58,020 two G-tetrad structures (11%) of 525,890 structures formed across the genome [42]. In a transcriptome-wide study of G4 formation with rG4-seq, Kwok et al. (2016) identified 639 two G-tetrad structures (16.6%) of 3,845 G4 structures formed [43].

In this paper, we identify sequences with the potential to form G4 on both DNA strands in each coding and non-coding region on all three manatee PV genomes. We find that the majority of sequences identified were capable of forming G4 structures with two rather than three G-tetrads, and we confirm the formation of G4 secondary structures from three of these sequences. We find several G4 in similar locations on all three PVs as well as several G4 in similar locations unique to the two PVs forming genital lesions. G4 were also located near putative E2 binding sites in non-coding regions on all PVs indicating a possible role in replication. Although G4 were found in all coding and non-coding regions, G4 were significantly enriched in the E2, E4, and L2 regions on all three genomes, suggesting that G4 are evolutionarily preserved in this region.

## Results

### G4 sequence distribution

The number of putative G4 sequences, broken down by DNA strand, genomic region, and TmPV genome, is displayed in Table 1. As described further in the Materials and methods section, the sequences were identified using the Quadparser algorithm [34], and longer sequences supported the development of more than one G4 at a time. As a result, in several regions the number of G4 possible was slightly higher than the number of sequences identified, and these values are displayed alongside the number of G4 sequences in Table 1. TmPV4 had the highest number of sequences identified, with 20 on the forward DNA strand and 17 on the reverse strand. Somewhat fewer sequences were identified on TmPV3 (13 forward, 11 reverse) and TmPV1 (14 forward, 15 reverse).

**Table 1:** The number of putative G4 sequences identified and the number of structures possible across different regions on forward and reverse DNA strands for each TmPV genome.

	Forward DNA Strand Number Sequences (Number Possible Structures)			Reverse DNA Strand Number Sequences (Number Possible Structures)		
Region	TmPV1	TmPV3	TmPV4	TmPV1	TmPV3	TmPV4
E6	-	1 (1)	-	-	-	-
E7	2 (2)	1 (1)	-	-	-	-
E1	5 (5)	3 (3)	4 (5)	-	-	-
E2	1 (1)	1 (1)	2 (2)	1 (1)	-	-
E1/E2	1 (1)	1 (1)	1 (1)	-	-	-
E2/E4	1 (1)	-	7 (12)	3 (5)	3 (6)	6 (9)
Total Early Region	10 (10)	7 (7)	14 (20)	4 (6)	3 (6)	6 (9)
L2	2 (4)	2 (4)	3 (3)	5 (6)	5 (6)	7 (7)
L1	2 (2)	3 (3)	2 (2)	4 (5)	3 (3)	3 (3)
Total Late Region	4 (6)	5 (7)	5 (5)	9 (11)	8 (9)	10 (10)
NCR	-	1 (1)	1 (3)	2 (2)	-	1 (1)
Total Genome	14 (16)	13 (15)	20 (28)	15 (19)	11 (15)	17 (20)

All identified G4 sequences, with one exception, are capable of forming G4 with only two G-tetrads. The exception to this pattern was found in the L2 region of TmPV1 where a sequence capable of forming a three G-tetrad structure on the forward DNA strand was identified. This sequence was embedded in a much longer sequence also capable of forming a two G-tetrad structure. The individual sequences along with sequence locations and sequence descriptors for putative G4 identified in each TmPV genome are available in S1, S2 and S3 Tables.

### G4 enriched in E2/E4/L2 regions on all TmPVs

For all TmPVs, the number of G4, measured as the number possible in a region, was greater than expected in the E2, E4, and L2 regions when compared to a random distribution of G4 in the region. For E2 and E4, this occurred primarily on the reverse DNA strand (E2: TmPV1, *p* = 0.0001; TmPV3, *p* = 0.0002; TmPV4, *p* = 0.0001; E4: TmPV1, *p* = 0.04; TmPV3, *p* = 0.0002; TmPV4, *p* = 0.0004). However, TmPV4 also showed enrichment on the forward DNA strand (E2: *p* = 0.0001; E4: *p* = 0.0001). For L2, enrichment occurred on both DNA strands (L2 forward: TmPV1, *p* = 0.02; TmPV3, *p* = 0.001; TmPV4, *p* = 0.03; L2 reverse: TmPV1, *p* = 0.03; TmPV3, *p* = 0.05; TmPV4, *p* = 0.02). Only TmPV4 demonstrated enrichment in the non-coding region (p = 0.0006). The number of observed G4, the number of random simulations with G4 greater than or equal to the observed G4, and the associated significance values are available in S4 Table for each DNA strand in each genomic region on each TmPV.

### Co-occurring G4 locations across TmPVs

**Fig 2:**
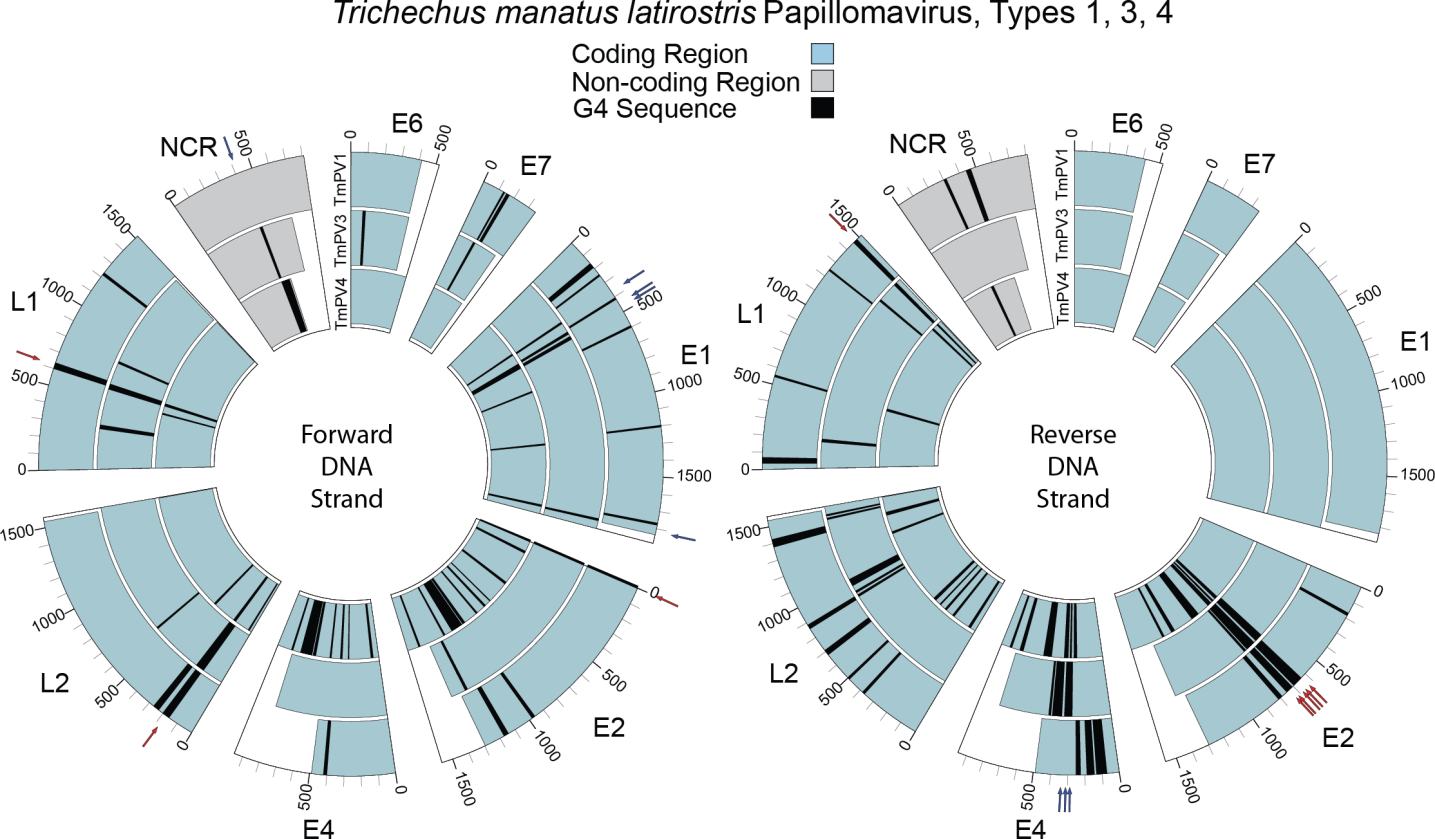
**Coding and non-coding regions aligned across TmPV1 (outer), TmPV3 (middle), and TmPV4 (inner) to illustrate G4 sequences occurring at the same location within regions on the forward (left) and reverse (right) DNA strands.** Blue arrows indicate G4 sequences at the same location on TmPV3 and TmPV4. Red arrows indicate G4 sequences at the same location on all three genomes.

To identify similar patterns in the distribution of G4 sequences across the three manatee PV genomes, the coding/non-coding regions were aligned at their starting locations, and G4 sequences with one or more nucleotides at the same distance from the beginning of the region were identified as occurring at the same location within a region. There were several regions with G4 sequences in the same location on all genomes. In Fig 2, on the forward DNA strand (left), G4 sequences were found in the same location in E2, L2, and L1. On the reverse DNA strand (Fig 2, right), G4 sequences were found in the same location in E2 and L1. There were also regions with G4 sequences occurring in the same location on TmPV3 and TmPV4 but not TmPV1. On the forward strand this pattern was found in E1 and NCR, and on the reverse strand, this pattern was found in E4.

### G4 located near putative E2 binding sites

**Fig 3:**
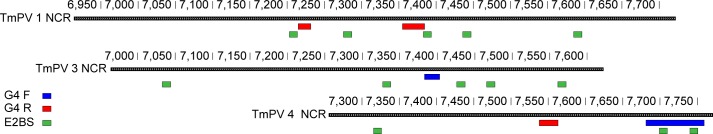
Location of G4 sequences in relation to putative E2 binding sites in the NCR region of each manatee papillomavirus. E2 binding sites are in green. G4 located on the forward DNA strand are in blue. G4 on the reverse DNA strand are in red.

On each PV, G4 sequences were identified in the non-coding region (NCR) where the origin of replication is located. Given the known role of G4 in replication [44, 45], these regions were searched for E2 binding sites to determine whether the G4 sequences might be positioned close to the origin of replication. E2 binding sites were identified using the consensus sequence ACCgNNNNcGGT, allowing some variation in the fourth and ninth nucleotide positions (lowercase g and c) with most variation occurring from nucleotide positions 5 through 8 [46]. S5 through S7 Tables list all sequences identified with the pattern ACCNNNNNNGGT. Nine of the 21 sequences identified have the more conservative consensus sequence of ACCGNNNNCGGT. The locations of consensus sequences identified in the NCR are displayed in Fig 3 along with the location of G4 in that region. On each PV genome, one or more G4 are located within 100 nt of a putative E2 binding site.

### Experimental confirmation of G4 formation in TmPV4 E2 sequences

**Fig 4:**
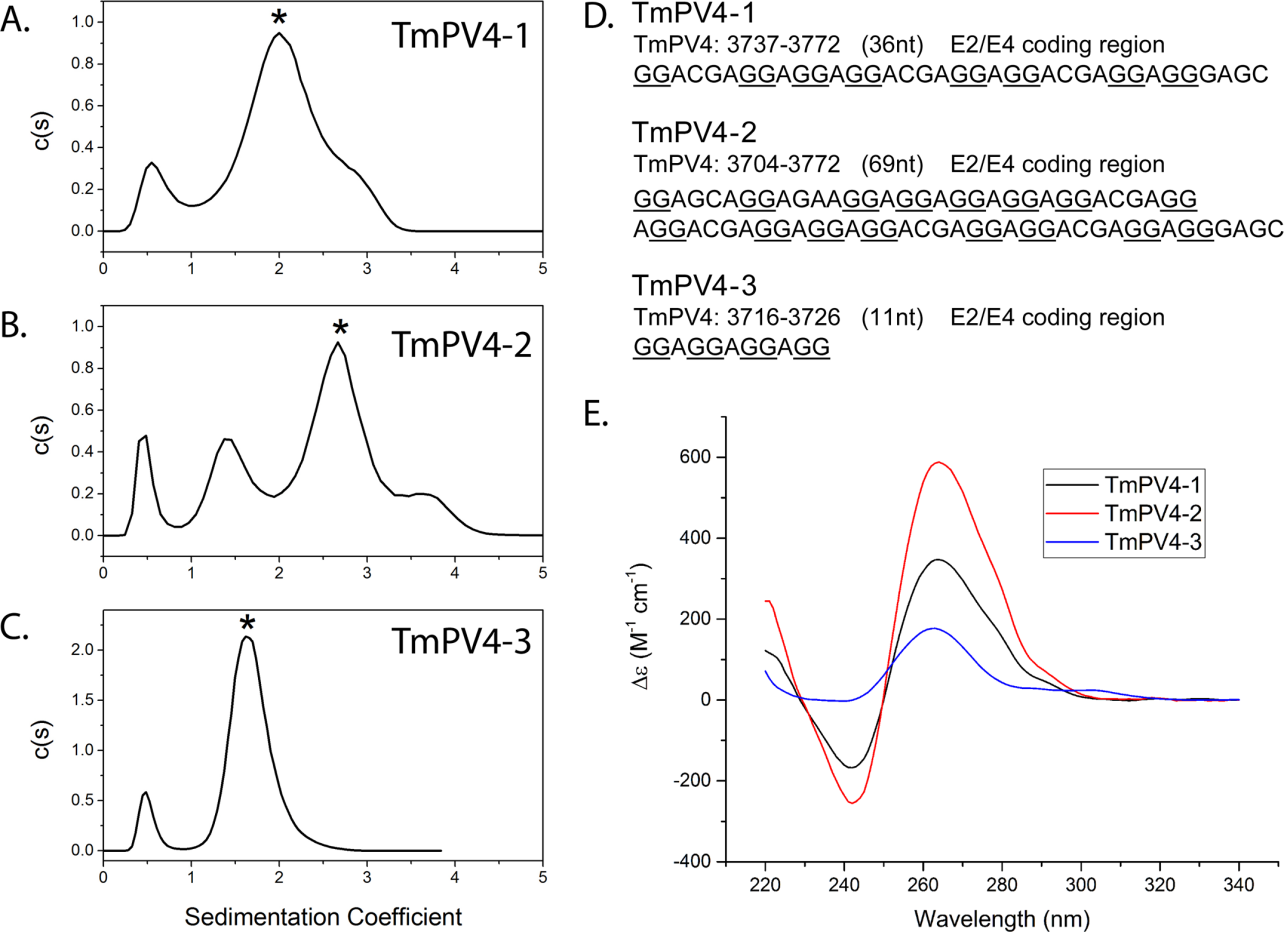
Analytical centrifugation of TmPV4 oligonucleotides (A, B, and C), oligonucleotide sequences (D), and CD spectra of TmPV4 oligonucleotides (E).

Using sequences identified by the bioinformatics analysis of the E2 region of TmPV4, three oligonucleotides were synthesized and experimentally tested for G4 formation (Fig 4D). Multiple well-established methods were used as criteria for G4 formation, using accepted standards in the field. These methods include analytical ultracentrifugation (AUC), circular dichroism (CD), thermal denaturation, and thioflavin T (ThT) binding. AUC is a venerable biophysical method that measures the absolute molecular weight (MW) and hydrodynamic shape (f/f_0_) of biomolecules. The frictional ratio f/f_0_ is the dimensionless ratio of the observed translational frictional coefficient to that of an equivalent spherical molecule of the same MW and density. It provides a measure of the asymmetry of the structure and shows definitively if a single-stranded oligonucleotide folds into a compact structure. The shape and amplitude of CD spectra provide characteristic signatures for the formation of G4 structures and for the number of G-tetrad stacks within a given structure. Thermal denaturation studies evaluate the stability of folded structures, and observed changes in CD spectra upon denaturation provide characteristic signatures for the transition from a folded G4 to an unstructured single strand. Finally, thioflavin T is a “light up” fluorescent probe for G4 formation. In the presence of known G4-forming sequences, a large increase in ThT fluorescence emission is observed, whereas in the presence of control duplexes and single strands, much lower emission intensity is seen. In addition to these accepted experimental criteria for G4 formation, molecular dynamics simulations were used to show that a plausible, stable, stereochemically feasible G4 can be constructed from the longest sequence studied, a structure that features two-tetrad G4 stacks.

Fig 4A–4C and Table 2 show the results of AUC experiments. Fig 4A–4C shows that each of the three oligonucleotides sequences displayed in Fig 4D folds into a single predominant species (indicated by asterisks) with a compact shape. TmPV4-1 (Fig 4A) shows a major peak at 2.15S, a frictional ratio of 1.36 and a measured MW of 12.1 kDA, a mass that is consistent with folding of the sequence into a unimolecular structure. TmPV4-1 shows slight (< 9%) heterogeneity with some faster sedimenting material. TmPV4-2 (Fig 4B) shows a major peak at 2.94S (MW = 22.5 kDa), again consistent with a unimolecular folded structure. TmPV4-2 is more heterogeneous with both faster and slower sedimenting species, most likely arising from the formation of small amounts of aggregated and partially unfolded structures, respectively. The fastest sedimenting peak in Fig 4B near 3.8S has a molecular weight of 42.7 kDa, indicating the formation of a minor amount of a bimolecular structure. TmPV4-3 (Fig 4C) shows a homogeneous peak at 1.78S (MW = 7.18 kDa). The measured mass is twice the MW of the 11 nt oligonucleotide showing unambiguously that a bimolecular structure has formed.

**Table 2:** Hydrodynamic properties of the major TmPV sequences*.

Sequence	MW (kDa)	s_20,w_	f/fo	Molecularity
TmPV4-1	12.1	1.96	1.36	monomer
TmPV4-2	22.5	2.79	1.58	monomer
TmPV4-3	7.18	1.8	1.09	dimer

*Determined using the non-interacting discrete species model in Sedfit.

Fortuitously, the structure of a 12 nt sequence identical to TmPV4-3, except for the addition of a 3’ terminal guanine residue, has been solved by NMR [47]. That structure shows that the oligonucleotide folds into a two-tetrad G4 structure that then dimerizes. The molecular weight we measure by AUC (Table 2) is fully consistent with the reported dimer structure. Further, the reported structure (1MYQ) can be used with the program HYDROPRO to compute an expected sedimentation coefficient. The calculated S-value obtained by calculation is 1.9 S, in excellent agreement with the observed value of 1.8 reported in Table 2, confirming that AUC reliably reports folding into a G4 structure.

Circular dichroism (CD) spectra strongly support G4 formation for all sequences. In Fig 4E, the spectra for each of the sequences shows a major positive peak at 260 nm along with a lesser negative peak at 240 nm in the presence of KCl. Such a spectral signature is accepted as characteristic of a parallel G4 topology [48, 49]. The amplitudes of the CD spectra in Fig 4E are proportional to the number of G-tetrads in each structure (R. D. Gray & J. B. Chaires, unpublished data) [50–53]. Oligonucleotides TmPV4-1 and TmPV4-2 have large amplitudes consistent with the formation of 4–5 and 7–8 G-tetrads, respectively. TmPV4-3 shows the lowest CD amplitude, consistent with the presence of 3–4 G-tetrads.

The results of thermal denaturation studies are shown in S1 Fig in the supplement. These results show that upon heating, the CD spectra of all three of the TmPV sequences studied is disrupted and changes from spectra characteristic of parallel G4 structures to spectra characteristic of unstructured single-strand oligonucleotides. The temperatures at the transition midpoint (T_m_) for TmPV4-1 and TmPV4-2 are 49.8 ± 0.2 and 52.1 ± 0.2 ^o^C, respectively. These T_m_ values demonstrate that stable unimolecular structures are formed by the oligonucleotides. The bimolecular TMPV4-3 has a T_m_ value of 82.7 ± 0.4 ^o^C, indicative of a very stable structure. The measured T_m_ values are well within the range of reported values for a wide variety of G4 structures determined under similar solution conditions [54].

Thioflavin T fluorescence was used to develop a “first-in-line assay to identify G4 formation” [55]. In the supplement, S2 Fig shows that ThT fluorescence is greatly enhanced in the presence of TmPV4-1 and TmPV4-2, indicating that they both fold into G4 structures. Enhancement of ThT fluorescence is less for TmPV4-3 (S2 Fig), but it has been noted that there are some outliers in the ThT assay. In particular, some G4 structures involving only two tetrads lead to a lower increase in ThT fluorescence.

## Discussion

The current study represents the first G4 sequences identified in PVs infecting a non-human animal. Prior to this, G4 sequences had been identified solely in PVs infecting humans [24]. The sequences identified in the current study consisted, almost exclusively, of sequences with two-guanine repeats, typically capable of forming structures comprised of only two G-tetrads. This differs from the consensus sequence G_≥3_N_1-7_G_≥3_N_1-7_G_≥3_N_1-7_G_≥3_ that is commonly used to search genomes for G-quadruplex forming potential. We found only one sequence capable of forming a three G-tetrad structure in one of three genomes in the L2 region.

While G4 structures containing only two G-tetrads are not commonly studied, they are not unprecedented. The well-known 15 nt thrombin binding aptamer (d(GGTTGGTGTGGTTGG)) forms an antiparallel “chair” quadruplex that is thermodynamically stable in potassium-containing solutions [40, 56]. The 12 nt GGA repeat sequence d(GGAGGAGGAGGA), nearly identical to our TmPV4-3, was shown by NMR to form a noncovalent, dimeric, two-quartet parallel structure in solution [35]. Finally, long GGA repeat sequences identified in the human c-*myb* gene have been found to arrest transcription [37]. Chemical footprinting methods were used to the show formation of multiple two-quartet G4 units within the proto-oncogene.

**Fig 5:**
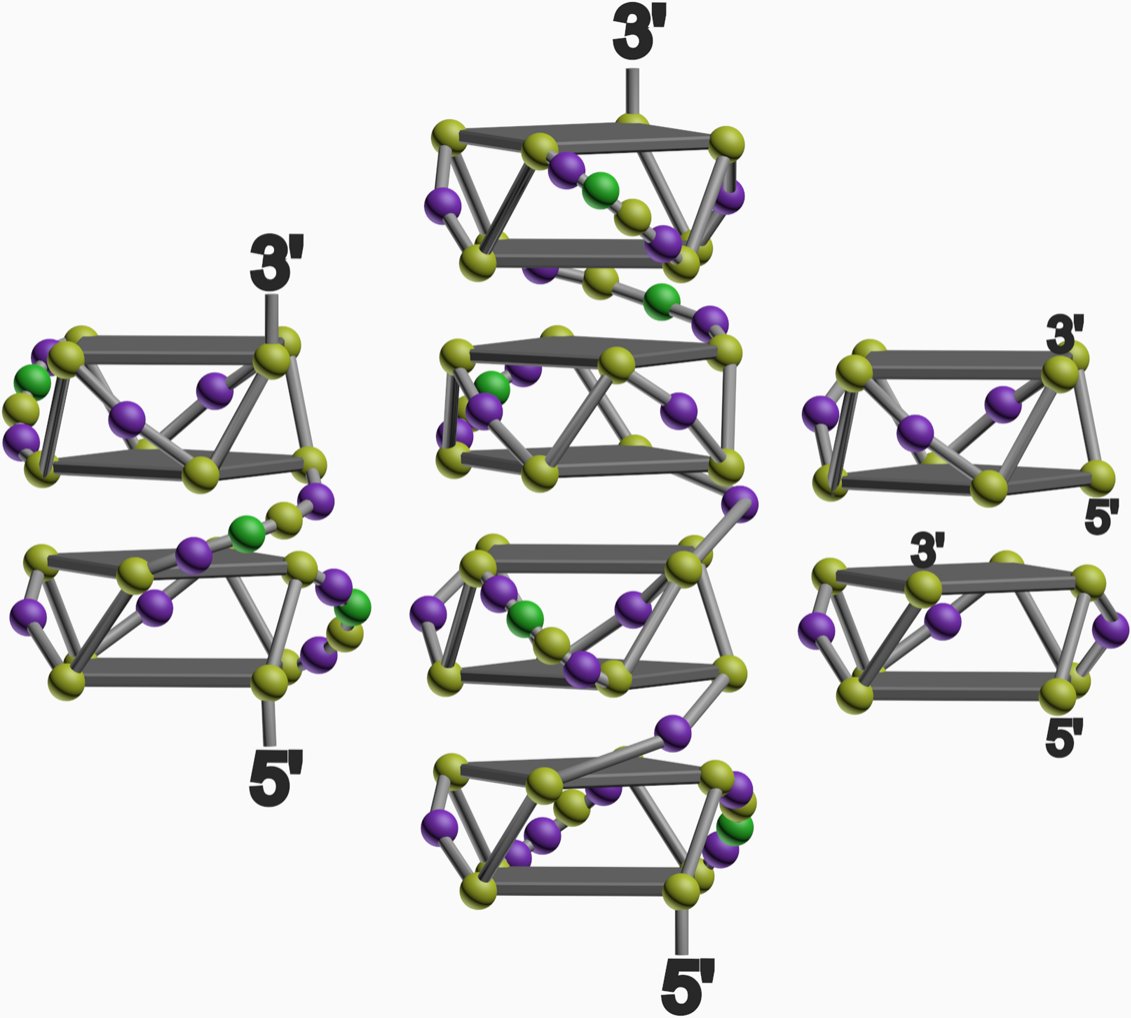
**Proposed structural conformation of TmPV4-1 (left), TmPV4-2 (middle), and TmPV4-3 (right).** Spheres represent guanine (yellow), adenine (purple), and cytosine (green) nucleotides.

The biophysical data shown in Fig 4, S1 Fig, S2 Fig, and Table 2 demonstrate that the three sequences tested from the E2 coding region of TmPV4 all form G-quadruplex structures. Although high-resolution NMR or x-ray crystallography would be required to determine definitive structures, we can suggest plausible models consistent with our biophysical data (see Fig 5). First, we consider TmPV4-3 because its sequence is nearly identical to the sequence underlying a published structure obtained by NMR [35, 57]. Matsugami and colleagues found that the sequence d(GGAGGAGGAGGA) folded into a parallel G-quadruplex structure with two stacked G-tetrads, that then dimerized, producing an assembly with four contiguous G-tetrads. Our AUC results on TmPV4-3 are fully consistent with the formation of a dimeric structure, and the amplitude of its CD spectrum indicates 3–4 stacked G-tetrads as would be expected from the reported structure.

Clearly, TmPV4-1 and TmPV4-2 are more complicated structures. Each forms a predominant monomeric structure as judged by their molecular weights (Table 2), and their CD signatures are consistent with the formation of a parallel G4 structure (Fig 4E). However, the CD spectra also indicate a greater number of G-tetrads than would be found in a single two G-tetrad structure. While it is possible to fold the TmPV4-1 and TmPV4-2 strands into a variety of G4 conformations, we believe the simplest and most probable structures are ones that feature stacked G4 units, in which each unit is folded in a parallel conformation with two G-tetrads, as shown in Fig 5. The observed frictional ratios of 1.36 for TmPV4-1 and 1.58 for TmPV4-2 (Table 2) indicate nonspherical, slightly elongated, hydrodynamic structures, consistent with the shapes of these multiquadruplex structural models. The stacking interactions between G4 units in the proposed models yield contiguous G-tetrad stacks that are consistent with the amplitudes of the CD spectra in Fig 4E. Definitive proof of our proposed models requires independent verification by high-resolution structural methods, but these models are consistent with the available biophysical evidence.

**Fig 6:**
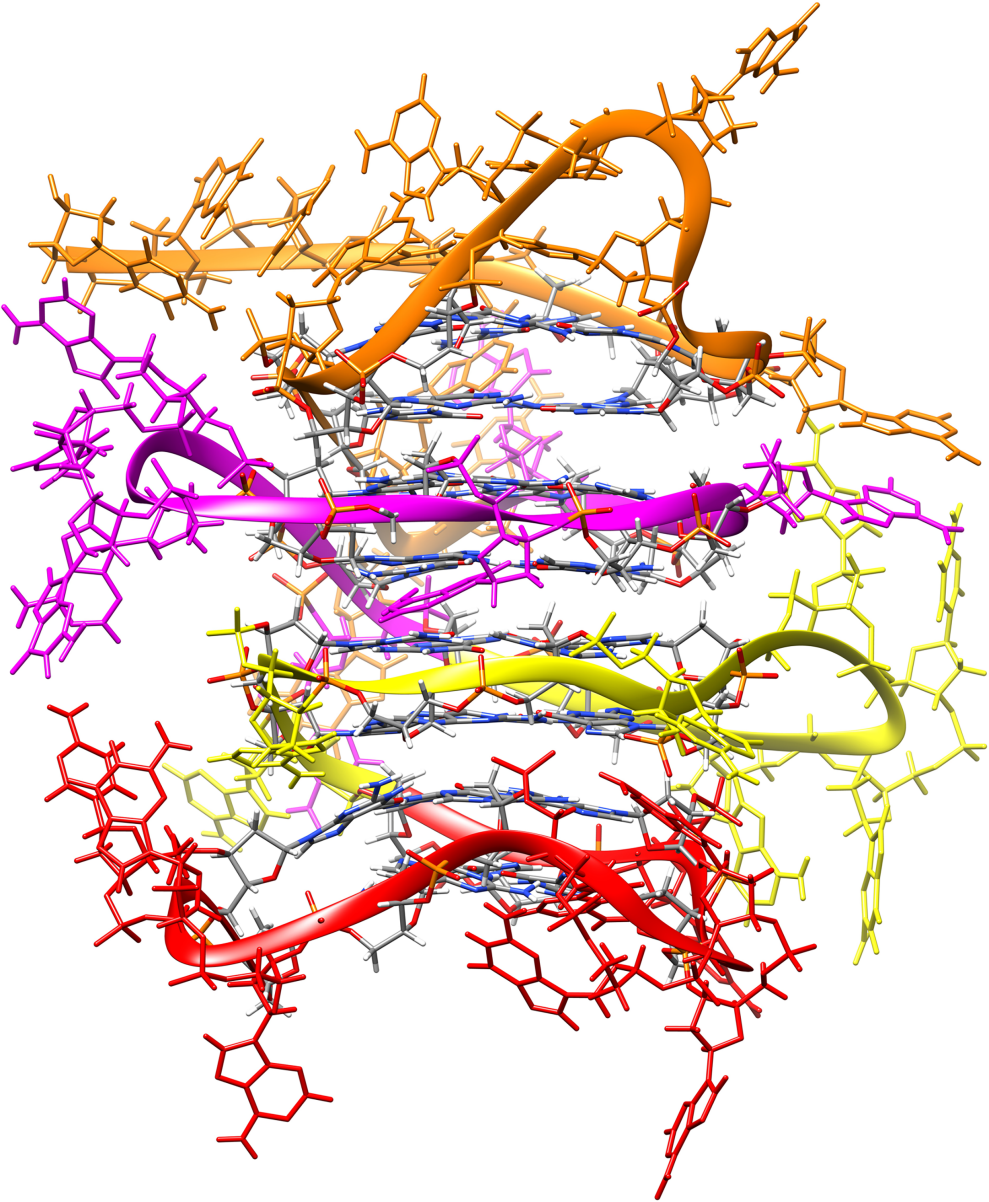
Molecular dynamics-derived model of the four stacked contiguous G-quadruplexes for sequence TmPV4-2. Individual G-quadruplex structures appear from top to bottom in orange, magenta, yellow, and red. G-tetrads are identified in elemental colors in Chimera **[88]**.

To explore further that such a model is feasible, molecular dynamics simulations were done to evaluate an atomic-level model for the TmPV4-2 sequence. The resultant model is shown in Fig 6, and is a detailed, stereochemically correct version of the schematic structure shown in Fig 5. The structure is stable over a 100 ns molecular dynamics production run, indicating that it represents one plausible model for the folded TmPV4-2 sequence. Again, NMR and x-ray crystallography studies will be necessary to determine the definitive G4 structure, but this model is fully consistent with the circular dichroism data.

It is interesting to note a consequence of the models shown in Fig 5. While an individual two G-tetrad structure might be only weakly stable, its stability could perhaps be enhanced by stacking interactions contributed by an adjacent G4 unit. Such a thermodynamic strategy might explain the proposed structures. With the short sequence in TmPV4-3, only a single two G-tetrad unit can form, which might then be stabilized by dimerization since no adjacent G4 unit, like those seen in TmPV4-1 and TmPV4-2, would be available for stacking.

Together, our computational and biophysical analyses suggest that the search for G4 in PVs, and perhaps all viruses, should be extended to include two G-tetrad structures. Our biophysical analysis indicates that this should include longer sequences capable of producing multiple stacked G4 units. In TmPVs these longer sequences are consistently found in the E2/E4 region on the reverse DNA strand, although they can be found elsewhere as well (See S8 Table).

Sequences capable of forming two G-tetrad structures are found throughout the human genome [41] and are known to form secondary structures [42, 43]. In at least one case, two-G-tetrad structures have been found to be more stable than three G-tetrad structures [58]. G4 with two G-tetrads are also known to form structures with different loop interactions and capping structures [59], supporting the notion that sequences capable of forming G4 are more variable than once thought. Furthermore, biological relevance for two G-tetrad structures has been found in bacteria [13] and two other viruses, HIV-1 and EBV [16, 22].

In the current study, sequences capable of forming structures with two G-tetrads are located more broadly across the genome than is seen in human PVs when searching for three G-tetrad structures. In the human viruses, three G-tetrad sequences were identified only in the E1 region on the forward DNA strand [24], whereas in manatee PVs we find two G-tetrad structures across all coding regions. Interestingly, the pattern we find on the reverse DNA strand for manatees is somewhat similar to that seen in human PVs; no sequences capable of forming G4 structures are found in E6, E7, or E1. If sequences supporting two G-tetrad structures were occurring merely by chance, one might expect to see a few in these areas as well. In manatee PVs, all subsequent regions contain two G-tetrad sequences. In human PVs, three G-tetrad sequences are found in all subsequent regions except L1.

On all three manatee PVs, G4 were identified at the same location in the L1 and L2 coding regions on the forward DNA strand and were enriched in the L2 region on both strands, perhaps indicating a conserved function for these sequences in the formation of the capsid proteins. New evidence suggests that G4 have a role in immune evasion through antigenic variation and viral silencing [60]. In *Neisseria gonorrhoeae*, a G4 structure is essential to variation in the surface protein pilin, allowing the bacteria to evade detection by the host immune system [14]. The proposed mechanism involves transcription of an sRNA from the G4 site that base-pairs with the complementary location on the opposite DNA strand, separating the two strands and allowing the formation of the G4. The G4 secondary structures are known to be mutagenic [61], and in HIV-1 and HIV-2, mutated capsids are known to affect the ability of dendritic cells to detect the virus [62].

G4 sequences were also enriched on the reverse DNA strand in the E2/E4 regions on all manatee PVs, suggesting an evolutionary advantage for G4 in this region. This is a significant location given that E2 is a negative regulator of E6/E7, two potential oncogenes. However, the formation of G4 on the template DNA strand would appear to serve simply as a block to the polymerase, inhibiting expression of the early genes, and it is not clear how this would provide an evolutionary advantage to the virus. Alternatively, G4 are known to have a mutagenic effect during replication [63], and G4 sequences in this area may provide some evolutionarily advantageous disruption of the sequence in this region.

G4 sequences were identified in non-coding areas on all three manatee PVs and were located near putative E2 binding sites, indicating a potential role in replication and/or transcription initiation. This would not be surprising given that G4 have been associated with origins of replication in mouse and humans [45, 64]. In a study of two vertebrate replicators, G4 were required for initiation of replication and determination of the replication start site [44].

On manatee PVs producing genital lesions, putative E2 binding sites were located in a pattern similar to that of genital human PVs [65]. One E2 binding site was located on the 5’ end of the non-coding region and at least two adjacent E2 binding sites were located on the 3’ end separated by fewer than 50 bases. The same pattern without the E2 binding site on the 5’ end is seen in the manatee PV producing cutaneous lesions. Interestingly, in each case a G4 sequence is located just upstream of the two adjacent 3’ E2 binding sites in an area that should be near the origin of replication. The location of these G4 within the PV promoter region may also be indicative of a role in transcriptional regulation. G4 are found in many promoter sites in humans and are known to regulate transcription [66]. In HPV16, two adjacent E2 binding sites in this area were found necessary for negative transcriptional regulation [67].

The distribution of G4 sequences within coding and non-coding regions across the viruses suggests that G4 may have a variety of regulatory roles. G4 sequences located on the forward DNA strand that are transcribed to mRNA could regulate splicing and translation of early and late genes by binding splicing factors or other related proteins or by serving to block the machinery necessary for each of these processes. In one other virus, Epstein-Barr virus, G4 have been found to inhibit translation of mRNA [22] making this an important avenue to explore in future studies. Similarly, on the reverse DNA strand, the formation of G4 could serve to inhibit transcription through blocking polymerase or binding proteins that enhance or inhibit transcription.

In all cases, G4 serve as valuable potential drug targets for viral control [6, 68]. In areas such as cancer research, much effort has been devoted to exerting regulatory control by identifying natural ligands [11, 69] and developing artificial ligands [70, 71] to promote G4 stabilization. Conversely, G4 destabilization can be achieved in the presence of a variety of helicases [72]. In terms of viral control, the focus has been on the therapeutic potential of G4-forming oligonucleotides, aptamers, and some success has been found in HIV [73], Hepatitis A [74], influenza virus [75], and severe acute respiratory syndrome coronavirus (SARS-CoV) [76]. More recently, viral control has been achieved by identifying ligands for regulating naturally occurring G4 in HIV-1 [15, 17–19, 21], EBV [22, 77], and HSV-1[23]. Given the greater likelihood of sequences representing two G-tetrad structures, even if structures do not naturally form *in vivo* from these sequences, the potential for control through ligand stabilization exists. Indeed, in high-throughput sequencing identification of G4 secondary structures, the number of two G-tetrad structures increases relative to the number produced with a potassium environment when adding the G4 stabilizing ligand pyridostatin [42, 43].

## Conclusion

This study provides further confirmation of the existence of G4 in PVs and extends previous work in human PVs by demonstrating the existence of non-canonical sequences, more broadly located across the genome, that are capable of forming G4 in non-human PVs. In longer sequences, these non-canonical, two G-tetrad structures appear capable of stacking as multiple G4 units. Distribution patterns that are indicative of G4 sequence conservation in specific coding regions support the notion that these structures regulate activities similar to those of G4 in other species. As regulatory structures, G4 offer potential drug targets for researchers interested in controlling disease processes. From an ecological perspective, given the threatened status of the Florida manatee and the concerns of scientists working to protect the health of this species, this research also provides an important first step in exploring the biological significance of these structures in this gentle aquatic mammal.

## Materials and methods

### Bioinformatics

The complete genomes for *Trichechus manatus* PVs (TmPV) 1, 3, and 4 were obtained from the NCBI nucleotide division of GenBank [78]. Information for accessing individual sequences can be found in Table 3.

**Table 3:** GenBank sequence information for TmPV genomes.

Accession no.	Description	Size
NC_006563.1	TmPV1	7722 bp
KP205502.1	TmPV3	7622 bp
KP205503.2	TmPV4	7771 bp

Putative G4 forming sequences were identified along each genome using the Quadparser algorithm [34]. For each genome, Quadparser was instructed to identify sequences capable of forming unimolecular structures with two or more G-tetrads. In each case, loop lengths were restricted to one to seven bases. Consequently, sequences took the following form: G_2+_, N_1-7_, G_2+_, N_1-7_, G_2+_, N_1-7_, G_2+_. Quadparser was instructed to search for G4 sequences on the forward DNA strand by searching for runs of guanine bases. However, G4 sequences on the reverse DNA strand were identified by searching for runs of cytosine, guanine’s complement.

**Fig 7:**
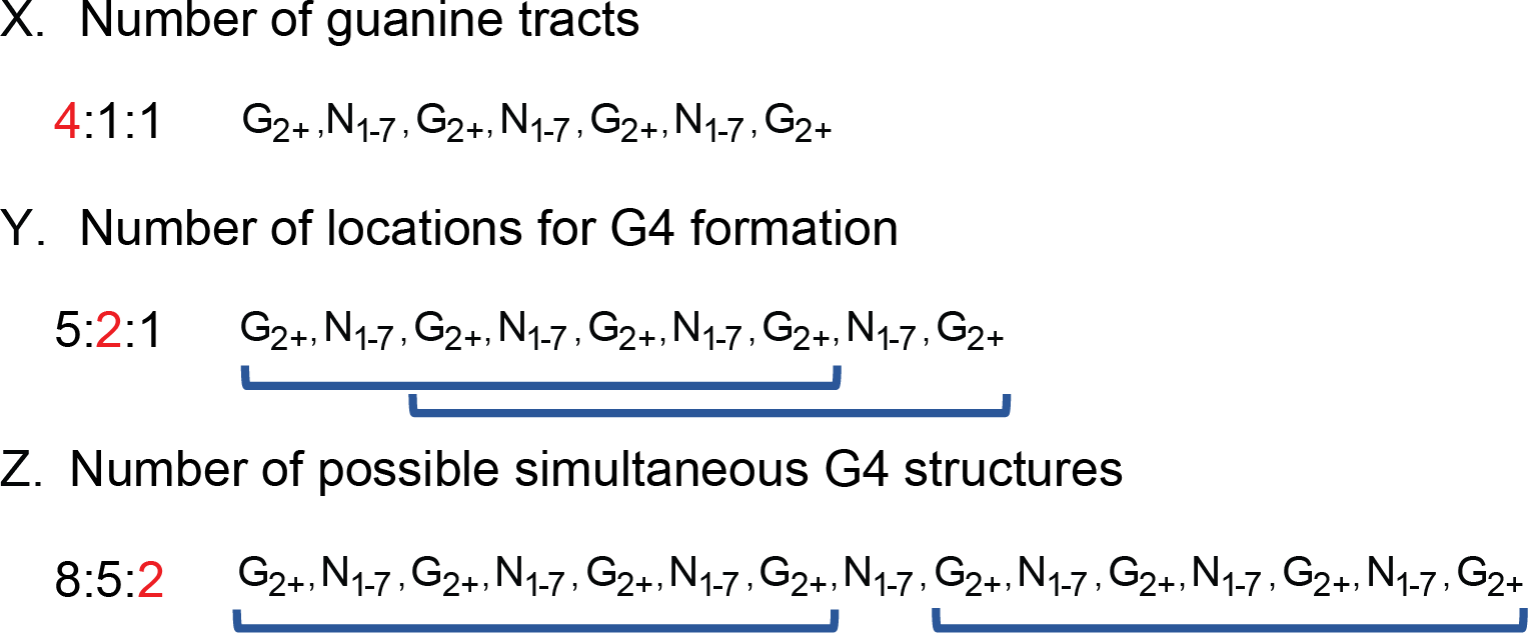
A description of the sequence codes provided by Quadparser for each putative G4 sequence. This Figure has been modified from [87].

Sequences identified by Quadparser generally vary in the number of guanine tracts. A G4 requires four guanine tracts to form. Therefore, sequences with five or more guanine tracks can form G4 at different locations in the sequence, and sequences with eight or more guanine tracts support the development of more than one G4 at a time. To highlight these variations, Quadparser provides a sequence descriptor in the form *x*:*y*:*z* (Fig 7), indicating the number of guanine tracts in the sequence (*x*), the number of locations at which a G4 could form (*y*), and the number of G4 possible at a given time within the sequence (*z*).

To establish values for the number of G4 sequences expected in each genomic region at random while taking GC content into account, a distribution of expected values was generated by randomly shuffling the nucleotides in each region. This was performed 10,000 times, each time counting the number of G4 sequences occurring at random. Significance values for the number of G4 sequences in each region were estimated as *P* = (*r* + 1)/(*n* + 1), where *r* is the number of random simulations in which the number of G4 sequences in a region was greater than or equal to the observed number of G4 sequences in that region and *n* is the number of random simulations [79, 80]. Significance levels were calculated for number of G4 sequences on each DNA strand in each region of the PV genomes.

Putative E2 binding sites were identified using the consensus sequence 5’-ACCgNNNNcGGT-3’ derived from a comprehensive study of human PVs [46]. The consensus sequence includes some variation in the fourth and ninth nucleotide positions with most variation occurring from nucleotide positions 5 through 8. To cast the widest net in searching for E2 binding sites in manatee PVs, positions 4 through 9 were allowed to vary over all nucleotides in the regular expression designed to search for these sequences.

### Oligonucleotides

Oligodeoxynucleotides (sequences in Table 4) were obtained as desalted, lyophilized solids and reconstituted in MilliQ water to give ~1 mM stock solutions. Oligos TmPV4-1, TmPV4-2 and TmPV4-3 were synthesized by Eurofins MWG Operon (Huntsville, AL) and oligos 1XAV and 1XAV-complement were from IDT, Coralville, IA. The oligos were reconstituted in MQ H_2_0 to give ~1 mM stock solutions. DNA strand concentrations were estimated from the absorbance at 260 nm of suitable dilutions into K^+^-free tBAP buffer (10 mM tetrabutyl ammonium phosphate, 1 mM EDTA, pH 7.0) in conjunction with extinction coefficients supplied by the manufacturer. Samples in KCl were annealed by heating in a 1-L boiling water bath for ~10 min followed by slow cooling to room temperature.

**Table 4:** Oligodeoxynucleotides used in this study.

Oligonucleotide	Sequence (5’ to 3’)	ε (mM^-1^ cm^-1^)	MW
TmPV4-1	d[GGACGAGGAGGAGGACGAGGAGGACGAGGAGGGAGC]	397.5	11453.4
TmPV4-2	d[GGAGCAGGAGAAGGAGGAGGAGGAGGACGAGGAGGACGAGGAGGAGGACGAGGAGGACGAGGAGGGAGC]	774.7	22045.3
TmPV4-3	d[GGAGGAGGAGG]	125.1	3511.3
1XAV	d[TGA GGG TGG GTA GGG TGG GTA A]	228.7	6991.6
1XAV-complement	d[TTA CCC ACC CTA CCC ACC CTC A]	191.5	6480.3

### Analytical ultracentrifugation

Sedimentation velocity experiments were carried out in a Beckman Coulter ProteomeLab XL-A analytical ultracentrifuge (Beckman Coulter Inc., Brea, CA) at 20°C and 50,000 rpm in standard 2 sector cells. Buffer density was determined on a Mettler/Paar Calculating Density Meter DMA 55A at 20.0 °C and viscosity was measured using an Anton Parr AMVn Automated Microviscometer at 20°C. A value of 0.55 ml/g was used for the DNA oligonucleotides as described [81]. Sedimentation coefficients, molecular weights and frictional ratios were determined using the program Sedfit (free software: www.analyticalultracentrifugation.com) using both the continuous c(s) distribution and the non-interacting discrete species models, and with the program Ultrascan III using the genetic algorithm and Monte Carlo features (free software: hhttp://uslims3.uthscsa.edu). The published structure for the sequence determined by NMR (pdb designation 1MYQ), which is identical to TmPV4-3 with 1 added 3’ G [35], was used to calculate the expected hydrodynamic properties for the two tetrad, dimeric quadruplex using the program winHYDROPRO10 (free software: http://leonardo.inf.um.es/macromol/programs/hydropro/hydropro.htm) with the residue/shell model [82].

### CD spectra

UV and CD spectra were measured at 25 °C in a stoppered 1-cm cuvette with a Jasco J-810 spectropolarimeter equipped with a programmable Peltier thermostated cuvette holder and magnetic stirrer as described [48]. Instrumental parameters were: 1.0 nm bandwidth, 2 s integration time, 200 nm/min scan rate, with four scans averaged. CD data were corrected by subtracting a buffer blank and normalized using the relationship ε_L_ − ε_R_ = Δε = θ/(32980·c·l), where θ is the observed ellipticity in millidegrees, c is the DNA strand concentration in mol·L^−1^, and l is the path length in cm.

### CD melts

Thermal denaturation was carried out on oligonucleotides annealed in tBAP/1 mM EDTA/50 mM KCl, pH 7.0. CD spectra were recorded from 340 nm to 220 nm over the temperature range of 20 °C to 98 °C at intervals of 2 °C using the above instrumental parameters. The temperature ramp was 4 °C/min, ± 0.05 °C equilibration tolerance and 60 s delay after equilibration before recording the spectrum. Melting was analyzed assuming a two-state folded (N) and unfolded (D) model. The normalized CD signal *y* at the CD maximum of 264 nm and temperature *T* was fit to Eq 1 [83, 84] to obtain midpoint (melting) temperature *T*_*m*_ and the van’t Hoff enthalpy *ΔH* for the transition (see Table 5).

**Table 5:** Thermodynamic parameters for thermal unfolding of TmPV4 oligonucleotides.

Oligonucleotide	*T*_*m*_ ± std dev of fit (°C)	*ΔH* ± std dev of fit (kcal/mol)
TmPV4-1	49.8 ± 0.2	54.7 ± 0.2
TmPV4-2	52.1 ± 0.2	63.5 ± 0.3
TmPV4-3	82.7 ± 0.4	63.1 ± 0.2

$$y=\frac{y_{N}+m_{N}T+\left(y_{D}+m_{D}T\right)e^{\Delta H/R(\frac{1}{T_{m}}-\frac{1}{T})}}{1+e^{\Delta H/R(\frac{1}{T_{m}}-\frac{1}{T})}}$$ Eq 1

In Eq 1, *y*_*N*_, *m*_*N*_, *y*_*D*_ and *m*_*D*_ are the intercepts and slopes of the (linear) pre- and post-transition baselines, respectively. The parameters *y*_*N*_, *m*_*N*_, *y*_*D*_, *m*_*D*_, *ΔH* and *T*_*m*_ were adjusted by non-linear least squares to give the best fit of the data to the Eq 1. The non-linear least squares routines in the program OriginPro 2016 (OriginLab Corporation, Northampton, MA) were used for data analysis.

### Thioflavin T fluorescence enhancement

Thioflavin T fluorescence is reported to be enhanced in the presence of quadruplex DNA but not in the presence of other common oligonucleotide structures [55]. To assess potential quadruplex formation, thioflavin T emission spectra were determined at 25 °C as described by de la Faverie et al. [55] with 0.5 μM thioflavin T (Sigma) and 1 μM oligodeoxynucleotide annealed in tBAP/1 mM EDTA/50 mM KCl, pH 7.0. Thioflavin T was excited at 412 nm (1 nm spectral band pass) and emission spectra were recorded from 420 to 620 nm (1 nm resolution, 2 nm spectral band pass) using a SpectraMax-3 fluorometer (Horiba Instruments, Edison, NJ). The integration time was 1 s and two spectra were averaged. The emission spectra were corrected by subtracting a buffer blank. The oligonucleotide 1XAV (a *c-myc* analog) was used as a positive control for fluorescence enhancement and its complementary (unfolded) DNA served as a negative control for non-specific fluorescence. The degree of fluorescence enhancement was calculated as the ratio of fluorescence intensities at the 488 nm emission maximum of the sample relative to the emission intensity of thioflavin T alone (F/F_0_).

### Molecular dynamics simulations

The molecular model of the four stacked contiguous quadruplexes for sequence TmPV4-2 d(GGAGCAGGAGAAGGAGGAGGAGGAGGACGAGGAGGACGAGGAGGAGGACGAGGAGGACGAGGAGGGAGC) was built based on the G-tetrads from the parallel stacked quadruplex 1KF1 [85]. The four quadruplex sequences were defined as GGAGCAGGAGAAGGAGG, GGAGGAGGACGAGG, GGACGAGGAGGAGG, and GGAGGACGAGGAGG with potassium ions placed centrally between tetrads and stacks. Loop regions were added manually in a parallel (double chain reversal) orientation. Molecular dynamics was performed using the AMBER suite of software [86]. The ff14SB force field was used and the system was solvated in a rectilinear box of TIP3P water molecules with 15 Å buffers and neutralizing K^+^ ions were added to the system using standard Leap rules. The system was heated and equilibrated using the following protocol: (i) minimize water and ions holding the DNA and tetrad coordinated potassium ions restrained (10 kcal/mol/Å), (ii) 25 ps MD (heating to 100 K) holding the DNA and tetrad coordinated potassium ions restrained (10 kcal/mol/Å), (iii) repeat step (i), (iv) minimize all atoms, (v) 25 ps MD (heating to 300 K) holding the DNA and tetrad coordinated potassium ions restrained (10 kcal/mol/Å), (vi) 5 ns MD (T = 300 K) equilibration holding the DNA and tetrad coordinated potassium ions restrained (10 kcal/mol/Å) to finish the equilibrium. A production run of 100 ns after the final equilibration step was carried out. Simulations were performed in the isothermal isobaric ensemble (P = 1 atm, T = 300 K) using sander and the GPU version of pmemd. Periodic boundary conditions and Particle-Mesh-Ewald algorithms were used. A 2.0 fs time step was used with bonds involving hydrogen atoms frozen using SHAKE.

## Supporting information

S1 Table Sequences, locations, and descriptors for putative G4 identified on TmPV1. Note that all sequences are identified on a reference genome. Thus, G4 sequences on the reverse DNA strand are identified by searching for C-tracts.
(PDF)

S2 Table Sequences, locations, and descriptors for putative G4 identified on TmPV3. Note that all sequences are identified on a reference genome. Thus, G4 sequences on the reverse DNA strand are identified by searching for C-tracts.
(PDF)

S3 Table Sequences, locations, and descriptors for putative G4 identified on TmPV4. Note that all sequences are identified on a reference genome. Thus, G4 sequences on the reverse DNA strand are identified by searching for C-tracts.
(PDF)

S4 Table The number of observed G4, the number of random simulations with G4 greater than or equal to the observed G4, and the associated significance values for each DNA strand in each genomic region on each TmPV along with the proportion of guanine and cytosine in each region. Cytosine content reflects guanine content on the reverse DNA strand.
(PDF)

S5 Table Putative E2 binding site sequences and locations on TmPV-1 along with the location and distance of the nearest putative G4 sequence. (PDF)

S6 Table Putative E2 binding site sequences and locations on TmPV-3 along with the location and distance of the nearest putative G4 sequence. (PDF)

S7 Table Putative E2 binding site sequences and locations on TmPV-4 along with the location and distance of the nearest putative G4 sequence. (PDF)

S8 Table Sequences, locations, and descriptors for G4 sequences capable of forming stacked G4 units. Note that all sequences are identified on a reference genome. Thus, G4 sequences on the reverse DNA strand are identified by searching for C-tracts. Guanine tracts are highlighted in red.
(PDF)

S1 Fig Temperature-dependent normalized CD spectra for TmPV4 oligonucleotides. Panels A, C and E depict the family of CD spectra for TmPV4-1, -2 and -3, respectively, recorded at 2-°C intervals between 20 °C and 98 °C. Panels B, D and E show the temperature dependence of the normalized CD signal at the maximum wavelength of 264 nm for each oligonucleotide. The points represent the experimental data and the lines show the best fit of the data to Eq 1. The resulting optimized thermodynamic parameters are summarized in Table 5. Conditions: 10 mM tBAP, 1 mM EDTA, 50 mM KCl, pH 7.0.
(PDF)

S2 Fig Thioflavin T fluorescence intensity given in counts/s (cps) in the absence of DNA and in the presence of TmPV4 oligonucleotides and controls 1XAV and 1XAV-complement. Panel A depicts the thioflavin T emission spectra of TmPV4-1, TmPV4-2, and 1XAV. Panel B shows the weak emission of thioflavin T in the absence of DNA as well as in the presence of TmPV4-3 and 1XAV-complement. Panel C shows the fold enhancement F/F_0_ of thioflavin T fluorescence in the presence of DNA (F) relative to that of the DNA-free emission intensity (F_0_). Conditions: 0.5 μM thioflavin T, 1 μM DNA strand, 10 mM tBAP, 1 mM EDTA, 50 mM KCl, pH 7.0, 25 °C.
(PDF)
