# Supplementary material for: Identification of G-quadruplex forming sequences in three manatee papillomaviruses
Source: PLoS One. 2018 Apr 9;13(4):e0195625. doi: 10.1371/journal.pone.0195625 (PMC5891072; doi:10.1371/journal.pone.0195625)
Supplement: S1 Table — Note that all sequences are identified on a reference genome. Thus, G4 sequences on the reverse DNA strand are identified by searching for C-tracts. (PDF) [file pone.0195625.s001.pdf]

S1 Table. **Sequences, locations, and descriptors for putative G4 identified on TmPV1.** Note that all sequences are identified on a reference genome. Thus, G4 sequences on the reverse DNA strand are identified by searching for C-tracts.

G4 Sequence Name*	Genome Start	Genome End	Sequence Code**	Sequence
TmPV1_1_E2_R	2707	2725	4:1:1	CCAGCCTATTCCAACAACC
TmPV1_2_E2/E4_R	3173	3235	11:8:2	CCAGACGGACCCGCACCCACTACCACCGGAGACCTATCCGAGCCATCCCCTGGATCCATCCCC
TmPV1_3_E2/E4_R	3246	3291	9:6:2	CCCCCACCGACACACCCGTCACCCATACCGAGGAGTCCATACCCCC
TmPV1_4_E2/E4_R	3326	3354	6:3:1	CCACCCACCAATACCGGGGGAACCACACC
TmPV1_5_L2_R	4249	4266	4:1:1	CCGGATATCCCGGCCACC
TmPV1_6_L2_R	4371	4386	4:1:1	CCTTCACCCCTCCACC
TmPV1_7_L2_R	4558	4592	7:4:1	CCACGTGCCACCAGCACGCCGGAAGGCCCTCCTCC
TmPV1_8_L2_R	4742	4769	6:3:1	CCACCCTACCAACCACTACCCCGCACCC
TmPV1_9_L2_R	5261	5308	8:5:2	CCACTGATACCAGCCCCCCTATACACCCTCTAACCCCACCTACAATCC
TmPV1_10_L1_R	5431	5467	8:5:2	CCTACCCCCCACCCCTGCCGCCCGCATCCTCAATACC
TmPV1_11_L1_R	5939	5952	4:1:1	CCCCCACCTCTGCC
TmPV1_12_L1_R	6635	6646	4:1:1	CCCCCTCCCTCC
TmPV1_13_L1_R	6851	6879	5:2:1	CCTCGTCCAGGCACCTCTACCTCTACCCC
TmPV1_14_NCR_R	7215	7232	4:1:1	CCCCCGCGGCACCTCACC
TmPV1_15_NCR_R	7355	7385	7:4:1	CCTTCAACCTGGCATCCCTCCACCTCCGACC
TmPV1_1_E7_F	554	564	4:1:1	GGTGGAGGAGG
TmPV1_2_E7_F	575	595	5:2:1	GGAAAAGGCGGAGGATAGTGG
TmPV1_3_E1_F	923	955	5:2:1	GGAAAGAGGCAGACAGGCATAAACGGGACTTGG
TmPV1_4_E1_F	1005	1016	4:1:1	GGGGGTGGGAGG
TmPV1_5_E1_F	1168	1181	4:1:1	GGTGGGGGGCAGGG
TmPV1_6_E1_F	1353	1366	4:1:1	GGATTGGGTGGCGG
TmPV1_7_E1_F	1959	1971	4:1:1	GGAGGGGGATTGG
TmPV1_8_E1/E2_F	2529	2543	5:2:1	GGAGGAGGAGGATGG
TmPV1_9_E2/E4_F	3501	3522	4:1:1	GGACATTGGGAAGGGGATCTGG
TmPV1_10_E2_F	3684	3714	7:4:1	GGAAGTGGTCAGGGTCGGGGGCTAAGGAGGG
TmPV1_11_L2_F	4039	4085	9:6:2	***GGTAGATTGGGTATTGGGACTGGGTCGGGTGCAGGGGGAAGTGGAGG

G4 Sequence Name*	Genome Start	Genome End	Sequence Code**	Sequence
TmPV1_12_L2_F	4114	4152	8:5:2	GGTGGCGCCAGGTTGGAGCCTGGCGGAGCAGTGGTCAGG
TmPV1_13_L1_F	5995	6032	7:4:1	GGATGGCGATATGGCGGATCTAGGCTTCGGTGCCATGG
TmPV1_14_L1_F	6610	6627	4:1:1	GGAGGATTGGCATATAGG

*The G4 sequence name consists of genome_number on genome_region_DNA strand.
** The number of G-tracts: number of G4 starting locations: number of G4 that can form simultaneously
***The sequence highlighted in red is the single sequence identified with the capability to form a G4 with three G-tetrads.
