# Supplementary material for: Identification of G-quadruplex forming sequences in three manatee papillomaviruses
Source: PLoS One. 2018 Apr 9;13(4):e0195625. doi: 10.1371/journal.pone.0195625 (PMC5891072; doi:10.1371/journal.pone.0195625)
Supplement: S2 Table — Note that all sequences are identified on a reference genome. Thus, G4 sequences on the reverse DNA strand are identified by searching for C-tracts. (PDF) [file pone.0195625.s002.pdf]

S2 Table. Sequences, locations, and descriptors for putative G4 identified on TmPV3. Note that all sequences are identified on a reference genome. Thus, G4 sequences on the reverse DNA strand are identified by searching for C-tracts.

G4 Sequence Name*	Genome Start	Genome End	Sequence Code**	Sequence
TmPV3_1_E2/E4_R	3163	3220	10:7:2	CCACACCCGACGACCTCCTCCCCGAAACCGAAGCCCACTGCCCTATCACCTCAACCCC
TmPV3_2_E2/E4_R	3237	3307	12:9:3	CCAGAACGGCCCGCCGGACCTGCCACCGCGTACACCGTTACCTACTCCCCTACCCCTGCAGCCGGAGCACC
TmPV3_3_E2/E4_R	3316	3333	5:2:1	CCACCCCCACTACCTGCC
TmPV3_4_L2_R	4793	4810	4:1:1	CCCCCCTGCAGCCTCCCC
TmPV3_5_L2_R	4825	4841	4:1:1	CCGCCCCTGACCCTGCC
TmPV3_6_L2_R	4882	4935	9:6:2	CCACTGCTACCAACCGTGTCCGGGTCAGCCGCCTTGGTACCCGTCCAGGCGTCC
TmPV3_7_L2_R	5388	5402	4:1:1	CCTCACCTGACCTCC
TmPV3_8_L2_R	5414	5427	4:1:1	CCCCCACCCTAGCC
TmPV3_9_L1_R	5648	5671	4:1:1	CCCCGCACCAATACCGTGTTTTCC
TmPV3_10_L1_R	6751	6767	4:1:1	CCTCCTGTACCTGATCC
TmPV3_11_L1_R	6906	6930	6:3:1	CCCCCTGCCGCCAAGTAAGCCCCCC
TmPV3_1_E6_F	85	106	4:1:1	GGAGGAAAACTTGGGACGTTGG
TmPV3_2_E7_F	539	555	4:1:1	GGAGGAGGAGTCGCAGG
TmPV3_3_E1_F	1075	1090	4:1:1	GGAGGGGGATAGTGGG
TmPV3_4_E1_F	1159	1178	4:1:1	GGTAGAGGGATATGGTTGGG
TmPV3_5_E1_F	1195	1220	7:4:1	GGGAGGGCAGGGGGGAAGGACGGAGG
TmPV3_6_E1/E2_F	2542	2559	4:1:1	GGACGAGGGAGAGGATGG
TmPV3_7_E2_F	3792	3812	5:2:1	GGTGAGGACGCTGGTGGCAGG
TmPV3_8_L2_F	4077	4141	12:9:3	GGGAATCTAGGGATAGGTACAGCAGGTGGGGGTGGGGGAAGATTTGGATATGGGGCCCTTGGCGG
TmPV3_9_L2_F	4518	4532	4:1:1	GGTGGGGGCTCTGGG
TmPV3_10_L1_F	5750	5780	6:3:1	GGGGATGTAGAGGTTTGGAGGTAGGCCGTGG
TmPV3_11_L1_F	6040	6080	7:4:1	GGGGATATGGGGGATATAGGTTTTGGGGCTATGGATTTCGG
TmPV3_12_L1_F	6236	6253	4:1:1	GGGCAGGCAAAATGGGGG
TmPV3_13_NCR_F	7383	7403	4:1:1	GGAATGGATTAGTGGAATTGG

* The G4 sequence name consists of genome_number on genome_region_DNA strand.

** The number of G-tracts: number of G4 starting locations: number of G4 that can form simultaneously
