# Supplementary material for: Identification of G-quadruplex forming sequences in three manatee papillomaviruses
Source: PLoS One. 2018 Apr 9;13(4):e0195625. doi: 10.1371/journal.pone.0195625 (PMC5891072; doi:10.1371/journal.pone.0195625)
Supplement: S3 Table — Note that all sequences are identified on a reference genome. Thus, G4 sequences on the reverse DNA strand are identified by searching for C-tracts. (PDF) [file pone.0195625.s003.pdf]

S3 Table. **Sequences, locations, and descriptors for putative G4 identified on TmPV4.** Note that all sequences are identified on a reference genome. Thus, G4 sequences on the reverse DNA strand are identified by searching for C-tracts.

G4 Sequence Name*	Genome Start	Genome End	Sequence Code**	Sequence
TmPV4_1_E2/E4_R	3273	3303	7:4:1	CCGCCGCCCCAGCCACCGCGCCCACAGTCCC
TmPV4_2_E2/E4_R	3323	3340	4:1:1	CCCTCCTCTGACCATCCC
TmPV4_3_E2/E4_R	3354	3393	6:3:1	CCCCAAGGACCCCATCGCCACGGCCGGCGGCCGGAGCACC
TmPV4_4_E2/E4_R	3509	3586	13:10:3	CCCGACCCTGGACCCGTCGCCTCAACCAGTGGGCCCGCCCCCAACCCCGAGACCAGTGACTCCGACACCTCAGACGCC
TmPV4_5_E2/E4_R	3777	3812	9:6:2	CCACCACCTCCACCACCTACACCAACCCCAACACCC
TmPV4_6_E2/E4_R	3887	3908	4:1:1	CCTACGGTCCTTCCTGGATTCC
TmPV4_7_L2_R	4270	4295	4:1:1	CCTGTAGACCCACAAATACCTGTTCC
TmPV4_8_L2_R	4456	4477	5:2:1	CCACCAGTGCAGCCAGACCCCC
TmPV4_9_L2_R	4548	4568	4:1:1	CCTTCCATTGTCCCGTTAACC
TmPV4_10_L2_R	4620	4633	5:2:1	CCCCCACCCCCACC
TmPV4_11_L2_R	4682	4711	7:4:1	CCTTCCATCCTCGGACCCTCCCATCCAACC
TmPV4_12_L2_R	5423	5444	4:1:1	CCCGAGTTCCACCCAATATACC
TmPV4_13_L2_R	5601	5633	6:3:1	CCGGAAGACCATAGCACCCCCAGAATACCTACC
TmPV4_14_L1_R	6271	6294	5:2:1	CCTCCCCCCGCAGGCGCCTGTCCC
TmPV4_15_L1_R	7143	7162	4:1:1	CCCCCTTGGCCGTAAATTCC
TmPV4_16_L1_R	7205	7221	4:1:1	CCTCTCCATCTACCTCC
TmPV4_17_NCR_R	7538	7563	6:3:1	CCATCCGCAACCGCCTCCGCTCTTCC
TmPV4_1_E1_F	1170	1186	4:1:1	GGAGGGGGATAGTGGGG
TmPV4_2_E1_F	1278	1327	9:6:2	GGTAGATAGGGGGGCTGTACGGCAGGGAGGCGGGGAACAACAGGGTGTGG
TmPV4_3_E1_F	1561	1576	4:1:1	GGAACGGAAGGGCGGG
TmPV4_4_E1_F	2064	2080	5:2:1	GGAGGGGGACTGGCGGG
TmPV4_5_E1/E2_F	2634	2654	5:2:1	GGAGGACGAGGGGGAAGATGG
TmPV4_6_E2_F	2748	2771	4:1:1	GGACTTTGGTCAGAAAGGAACAGG
TmPV4_7_E2/E4_F	3087	3110	5:2:1	GGAGAAAGGTGGAGGGTGCAGTGG
TmPV4_8_E2/E4_F	3312	3322	4:1:1	GGTGGGGGCGG
TmPV4_9_E2/E4_F	3375	3395	5:2:1	GGCCGGCGGCCGGAGCACCGG
TmPV4_10_E2/E4_F	3477	3493	4:1:1	GGCACCCGGGGGACGGG

G4 Sequence Name*	Genome Start	Genome End	Sequence Code**	Sequence
TmPV4_11_E2/E4_F	3623	3650	5:2:1	GGAGCCCGGTCCAGAGGAGGTAGA GGGG
TmPV4_12_E2/E4_F	3659	3769	24:21:6	GGGGGCCGAGGCAGAGGAGGCAGC GGCAGGGCCCAAGGGCACCGAGGA GCAGGAGAAGGAGGAGGAGGAGGA CGAGGAGGACGAGGAGGAGGACGA GGAGGACGAGGAGGG
TmPV4_13_E2/E4_F	3840	3857	4:1:1	GGCTGCACGGCGAGGGGG
TmPV4_14_E2_F	4093	4111	4:1:1	GGGGGAAGGGCCTAGTGGG
TmPV4_15_L2_F	4243	4257	4:1:1	GGGCTACGGTGGAGG
TmPV4_16_L2_F	4395	4421	6:3:1	GGTTCCGGTGGGGGTGGACGTTTAG GG
TmPV4_17_L2_F	4638	4657	5:2:1	GGCGGAGCGGGTCTCGGTGG
TmPV4_18_L1_F	6248	6263	4:1:1	GGGATAAGGTGGACGG
TmPV4_19_L1_F	6328	6353	4:1:1	GGCGACATGGGTGATATAGGGTTCG G
TmPV4_20_NCR_F	7681	7759	14:11:3	GGTGGGTACCAGGTGGGTACCGGGT GCGGTCTTCGGCGGGAGCCCCTGGC GGTCGGGCAACCGGGAACGGTCGTG CAGG

* The G4 sequence name consists of genome_number on genome_region_DNA strand.
** The number of G-tracts: number of G4 starting locations: number of G4 that can form simultaneously
