# Supplementary material for: Identification of G-quadruplex forming sequences in three manatee papillomaviruses
Source: PLoS One. 2018 Apr 9;13(4):e0195625. doi: 10.1371/journal.pone.0195625 (PMC5891072; doi:10.1371/journal.pone.0195625)
Supplement: S4 Table — Cytosine content reflects guanine content on the reverse DNA strand. (PDF) [file pone.0195625.s004.pdf]

S4 Table. **The number of observed G4, the number of random simulations with G4 greater than or equal to the observed G4, and the associated significance values for each DNA strand in each genomic region on each TmPV along with the proportion of guanine and cytosine in each region.** Cytosine content reflects guanine content on the reverse DNA strand.

PV	Region	Proportion Guanine	Number G4 Observed Forward Strand	Number of Random Simulations with G4 >= Observed (Forward)	P Value (Forward)	Proportion Cytosine	Number G4 Observed Reverse Strand	Number of Random Simulations with G4 >= Observed (Reverse)	P Value (Reverse)
TmPV1	E6	0.24	0	10,000	1	0.18	0	10,000	1
TmPV1	E7	0.27	2	1,010	0.1011	0.17	0	10,000	1
TmPV1	E1	0.24	6	60	0.0061**	0.16	0	10,000	1
TmPV1	E2	0.27	2	6,587	0.6587	0.23	6	0	0.0001***
TmPV1	E4	0.27	1	5,500	0.5500	0.33	5	407	0.0408*
TmPV1	L2	0.23	4	210	0.0211*	0.27	6	298	0.0299*
TmPV1	L1	0.20	2	559	0.0560	0.24	5	77	0.0078**
TmPV1	NCR	0.20	0	10,000	1	0.22	2	658	0.0659
TmPV3	E6	0.28	1	5,955	0.5955	0.17	0	10,000	1
TmPV3	E7	0.27	1	3,833	0.3834	0.22	0	10,000	1
TmPV3	E1	0.25	4	1,674	0.1675	0.15	0	10,000	1
TmPV3	E2	0.25	1	8,168	0.8168	0.23	6	1	0.0002***
TmPV3	E4	0.26	0	10,000	1	0.26	6	1	0.0002***
TmPV3	L2	0.21	4	10	0.0011**	0.27	6	538	0.0539*
TmPV3	L1	0.19	3	16	0.0017**	0.26	3	3,263	0.3264
TmPV3	NCR	0.22	1	2,840	0.2841	0.24	0	10,000	1
TmPV4	E6	0.25	0	10,000	1	0.19	0	10,000	1
TmPV4	E7	0.32	0	10,000	1	0.23	0	10,000	1
TmPV4	E1	0.25	6	145	0.0146*	0.18	0	10,000	1
TmPV4	E2	0.29	14	0	0.0001***	0.26	9	0	0.0001***
TmPV4	E4	0.30	12	0	0.0001***	0.30	9	3	0.0004***
TmPV4	L2	0.22	3	269	0.0270*	0.28	7	243	0.0244*
TmPV4	L1	0.21	2	1,063	0.1064	0.24	3	1,293	0.1294
TmPV4	NCR	0.22	3	5	0.0006***	0.27	1	6,415	0.6415

*p <= 0.05, **p < 0.01, ***p < 0.001
