# Supplementary material for: Identification of G-quadruplex forming sequences in three manatee papillomaviruses
Source: PLoS One. 2018 Apr 9;13(4):e0195625. doi: 10.1371/journal.pone.0195625 (PMC5891072; doi:10.1371/journal.pone.0195625)
Supplement: S5 Table — (PDF) [file pone.0195625.s005.pdf]

**S5 Table. Putative E2 binding site sequences and locations on TmPV 1 along with the location and distance of the nearest putative G4 sequence.**

| Region | Sequence | Genomic Position | Genomic Position of Nearest Upstream G4 (Distance) | Nearest Upstream G4 Sequence | Genomic Position of Nearest Downstream G4 (Distance) | Nearest Downstream G4 Sequence |
|---|---|---|---|---|---|---|
| NCR | ACC**CATGG**CGGT | 7203 | 6851 (-352) | CCTCGTCCAGGCACCTCTACCTCTACCCC | 7215 (+12) | CCCCCGCGGCACCTCACC |
| NCR | ACCG**CCTT**CGGT* | 7276 | 7215 (-61) | CCCCCGCGGCACCTCACC | 7355 (+79) | CCTTCAACCTGGCATCCCTCCACCTCCGACC |
| NCR | ACCG**TCTCG**GGT | 7383 | 7355 (-28) | CCTTCAACCTGGCATCCCTCCACCTCCGACC | - | |
| NCR | ACC**TAATGA**GGT | 7436 | 7355 (-81) | CCTTCAACCTGGCATCCCTCCACCTCCGACC | - | |
| NCR | ACCG**GATTA**GGT | 7585 | 7355 (-230) | CCTTCAACCTGGCATCCCTCCACCTCCGACC | - | |

*Conservative search sequence ACCGNNNNCGGT; Variable nucleotide positions are highlighted in red bold.
