# Supplementary material for: Identification of G-quadruplex forming sequences in three manatee papillomaviruses
Source: PLoS One. 2018 Apr 9;13(4):e0195625. doi: 10.1371/journal.pone.0195625 (PMC5891072; doi:10.1371/journal.pone.0195625)
Supplement: S6 Table — (PDF) [file pone.0195625.s006.pdf]

S6 Table. Putative E2 binding site sequences and locations on TmPV 3 along with the location and distance of the nearest putative G4 sequence.

Region	Sequence	Genomic Position	Genomic Position of Nearest Upstream G4 (Distance)	Nearest Upstream G4 Sequence	Genomic Position of Nearest Downstream G4 (Distance)	Nearest Downstream G4 Sequence
E1	ACCG**ATGTT**GGT	2344	1195 (-1149)	GGGAGGGCAGGGGGGAAGGACGGAGG	2542 (+198)	GGACGAGGGAGAGGATGG
E4	ACCG**CTGGA**GGT	3576	3316 (-260)	CCACCCCCACTACCTGCC	3792 (+216)	GGTGAGGACGCTGGTGGCAGG
NCR	ACCG**TAAC**CGGT*	7033	6906 (-127)	CCCCCTGCCGCCAAGTAAGCCCCCC	7383 (+350)	GGAATGGATTAGTGGAATTGG
NCR	ACCG**TTTGT**GGT	7327	6906 (-421)	CCCCCTGCCGCCAAGTAAGCCCCCC	7383 (+56)	GGAATGGATTAGTGGAATTGG
NCR	ACCG**TTCC**CGGT*	7426	7383 (-43)	GGAATGGATTAGTGGAATTGG	-	
NCR	ACCG**GGAG**CGGT*	7466	7383 (-83)	GGAATGGATTAGTGGAATTGG	-	
NCR	ACCG**TTAGG**GGT	7561	7383 (-178)	GGAATGGATTAGTGGAATTGG	-	

*Conservative search sequence ACCGNNNNCGGT; Variable nucleotide positions are highlighted in red bold.
