# Supplementary material for: Identification of G-quadruplex forming sequences in three manatee papillomaviruses
Source: PLoS One. 2018 Apr 9;13(4):e0195625. doi: 10.1371/journal.pone.0195625 (PMC5891072; doi:10.1371/journal.pone.0195625)
Supplement: S7 Table — (PDF) [file pone.0195625.s007.pdf]

S7 Table. Putative E2 binding site sequences and locations on TmPV 4 along with the location and distance of the nearest putative G4 sequence.

Region	Sequence	Genomic Position	Genomic Position of Nearest Upstream G4 (Distance)	Nearest Upstream G4 Sequence	Genomic Position of Nearest Downstream G4 (Distance)	Nearest Downstream G4 Sequence
E6	ACCG**GGTG**CGGT*	44	-		1170 (+1126)	GGAGGGGGATAGTGGGG
E6	ACC**AATAT**CGGT	320	-		1170 (+850)	GGAGGGGGATAGTGGGG
E1	ACCG**CTATT**GGT	2439	2064 (-375)	GGAGGGGGACTGGCGGG	2634 (+195)	GGAGGACGAGGGGGAAGATGG
E4	ACC**CAGGCG**GGT	3809	3777 (-32)	CCACCACCTCCACCACCTACACCAACCCCAACACCC	3840 (+31)	GGCTGCACGGCGAGGGGG
L2	ACCG**GTTC**CGGT*	4392	4270 (-122)	CCTGTAGACCCACAAATACCTGTTCC	4395 (+3)	GGTTCCGGTGGGGGTGGACGTTTAGGG
L2	ACC**CCCAGT**GGT	4472	4456 (-16)	CCACCAGTGCAGCCAGACCCCC	4548 (+76)	CCTTCCATTGTCCCGTTAACC
NCR	ACCG**CCAG**CGGT*	7315	7205 (-110)	CCTCTCCATCTACCTCC	7538 (+223)	CCATCCGCAACCGCCTCCGCTCTTCC
NCR	ACCG**GGTG**CGGT*	7699	7681 (-18)	GGTGGGTACCAGGTGGGTACCGGGTGCGGTCTTCGGCGGGAGCCCCTGGCGGTCGGGCAACCGGGAACGGTCGTGCAGG	-	
NCR	ACCG**GGAA**CGGT*	7740	7681 (-59)	GGTGGGTACCAGGTGGGTACCGGGTGCGGTCTTCGGCGGGAGCCCCTGGCGGTCGGGCAACCGGGAACGGTCGTGCAGG	-	

*Conservative search sequence ACCGNNNNCGGT; Variable nucleotide positions are highlighted in red bold.
