# Supplementary material for: Identification of G-quadruplex forming sequences in three manatee papillomaviruses
Source: PLoS One. 2018 Apr 9;13(4):e0195625. doi: 10.1371/journal.pone.0195625 (PMC5891072; doi:10.1371/journal.pone.0195625)
Supplement: S8 Table — Note that all sequences are identified on a reference genome. Thus, G4 sequences on the reverse DNA strand are identified by searching for C-tracts. Guanine tracts are highlighted in red. (PDF) [file pone.0195625.s008.pdf]

S8 Table. Sequences, locations, and descriptors for G4 sequences capable of forming stacked units. Note that all sequences are identified on a reference genome. Thus, G4 sequences on the reverse DNA strand are identified by searching for C-tracts. Guanine tracts are highlighted in red.

*G4 Sequence Name	Genome Start-End	Possible Stacked Units	Sequence
TmPV1_2_E2/E4_R	3173-3235	2	CCAGACGGACCCGCACCCACTACCACCGGAGACCTA TCCGAGCCATCCCCTGGATCCATCCCC
TmPV1_3_E2/E4_R	3246-3291	2	CCCCCACCGACACACCCGTCACCCATACCGAGGAGT CCATACCCCC
TmPV1_9_L2_R	5261-5308	2	CCACTGATACCAGCCCCCCTATACACCCTCTAACCCC ACCTACAATCC
TmPV1_10_L1_R	5431-5467	2	CCTACCCCCCACCCCTGCCGCCCGCATCCTCAATACC
TmPV1_11_L2_F	4039-4085	2	GGTAGATTGGGTATTGGGACTGGGTCGGGTGCAGGG GGAAGTGGAGG
TmPV1_12_L2_F	4114-4152	2	GGTGGCGCCAGGTTGGAGCCTGGCGGAGCAGTGGTC AGG
TmPV3_1_E2/E4_R	3163-3220	2	CCACACCCGACGACCTCCTCCCCGAAACCGAAGCCC ACTGCCCTATCACCTCAACCCC
TmPV3_2_E2/E4_R	3237-3307	3	CCAGAACGGCCCGCCGGACCTGCCACCGCGTACACC GTTACCTACTCCCCTACCCCTGCAGCCGGAGCACC
TmPV3_6_L2_R	4882-4935	2	CCACTGCTACCAACCGTGTCCGGGTCAGCCGCCTTG GTACCCGTCCAGGCGTCC
TmPV3_8_L2_F	4077-4141	3	GGGAATCTAGGGATAGGTACAGCAGGTGGGGGTGG GGGAAGATTTGGATATGGGGCCCTTGGCGG
TmPV4_4_E2/E4_R	3509-3586	3	CCCGACCCTGGACCCGTCGCCTCAACCAGTGGGCCC GCCCCCAACCCCGAGACCAGTGACTCCGACACCTCA GACGCC
TmPV4_5_E2/E4_R	3777-3812	2	CCACCACCTCCACCACCTACACCAACCCCAACACCC
TmPV4_2_E1_F	1278-1327	2	GGTAGATAGGGGGGCTGTACGGCAGGGAGGCGGGG AACAACAGGGTGTGG
TmPV4_12_E2/E4_F	3659-3769	6	GGGGGCCGAGGCAGAGGAGGCAGCGGCAGGGCCCA AGGGCACCGAGGAGCAGGAGAAGGAGGAGGAGGAG GACGAGGAGGACGAGGAGGAGGACGAGGAGGACGA GGAGGG
TmPV4_20_NCR_F	7681-7759	3	GGTGGGTACCAGGTGGGTACCGGGTGCGGTCTTCGG CGGGAGCCCCTGGCGGTCGGGCAACCGGGAACGGTC GTGCAGG

* The G4 sequence name consists of genome_number on genome_region_DNA strand.
