# Supplementary material for: Identification of G-quadruplex forming sequences in three manatee papillomaviruses
Source: PLoS One. 2018 Apr 9;13(4):e0195625. doi: 10.1371/journal.pone.0195625 (PMC5891072; doi:10.1371/journal.pone.0195625)
Supplement: S1 Fig — Panels A, C and E depict the family of CD spectra for TmPV4-1, -2 and -3, respectively, recorded at 2-°C intervals between 20 °C and 98 °C. Panels B, D and E show the temperature dependence of the normalized CD signal at the maximum wavelength of 264 nm for each oligonucleotide. The points represent the experimental data and the lines show the best fit of the data to Eq 1. The resulting optimized thermodynamic parameters are summarized in Table 5. Conditions: 10 mM tBAP, 1 mM EDTA, 50 mM KCl, pH 7.0. (PDF) [file pone.0195625.s009.pdf]

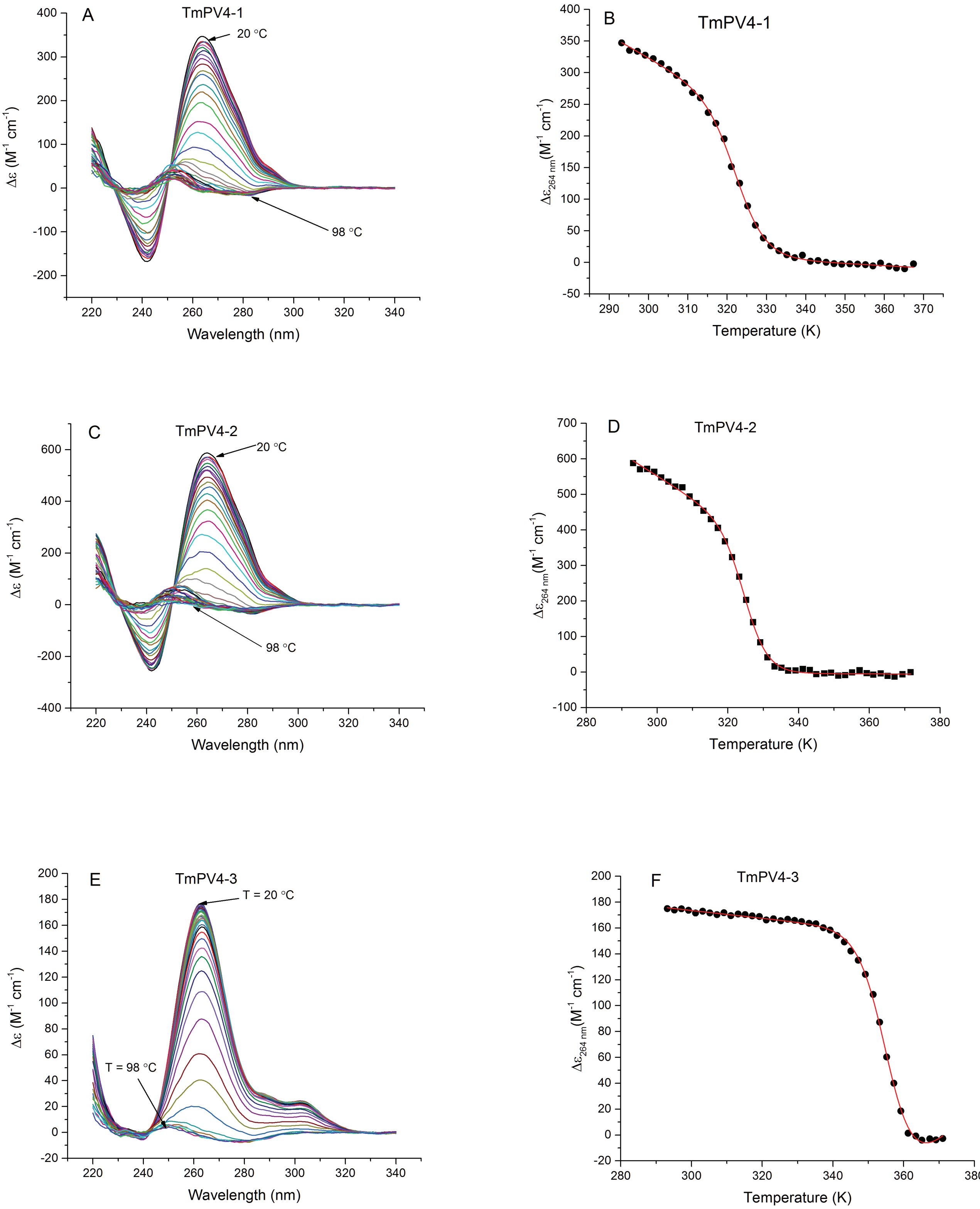

S1 Fig. Temperature-dependent normalized CD spectra for TmPV4 oligonucleotides. Panels A, C and E depict the family of CD spectra for TmPV4-1, -2 and -3, respectively, recorded at 2-°C intervals between 20 °C and 98 °C. Panels B, D and E show the temperature dependence of the normalized CD signal at the maximum wavelength of 264 nm for each oligonucleotide. The points represent the experimental data and the lines show the best fit of the data to Eq. 1. The resulting optimized thermodynamic parameters are summarized in Table 5. Conditions: 10 mM tBAP, 1 mM EDTA, 50 mM KCl, pH 7.0.
