# Supplementary material for: Identification of G-quadruplex forming sequences in three manatee papillomaviruses
Source: PLoS One. 2018 Apr 9;13(4):e0195625. doi: 10.1371/journal.pone.0195625 (PMC5891072; doi:10.1371/journal.pone.0195625)
Supplement: S2 Fig — Panel A depicts the thioflavin T emission spectra of TmPV4-1, TmPV4-2, and 1XAV. Panel B shows the weak emission of thioflavin T in the absence of DNA as well as in the presence of TmPV4-3 and 1XAV-complement. Panel C shows the fold enhancement F/F0 of thioflavin T fluorescence in the presence of DNA (F) relative to that of the DNA-free emission intensity (F0). Conditions: 0.5 μM thioflavin T, 1 μM DNA strand, 10 mM tBAP, 1 mM EDTA, 50 mM KCl, pH 7.0, 25 °C. (PDF) [file pone.0195625.s010.pdf]

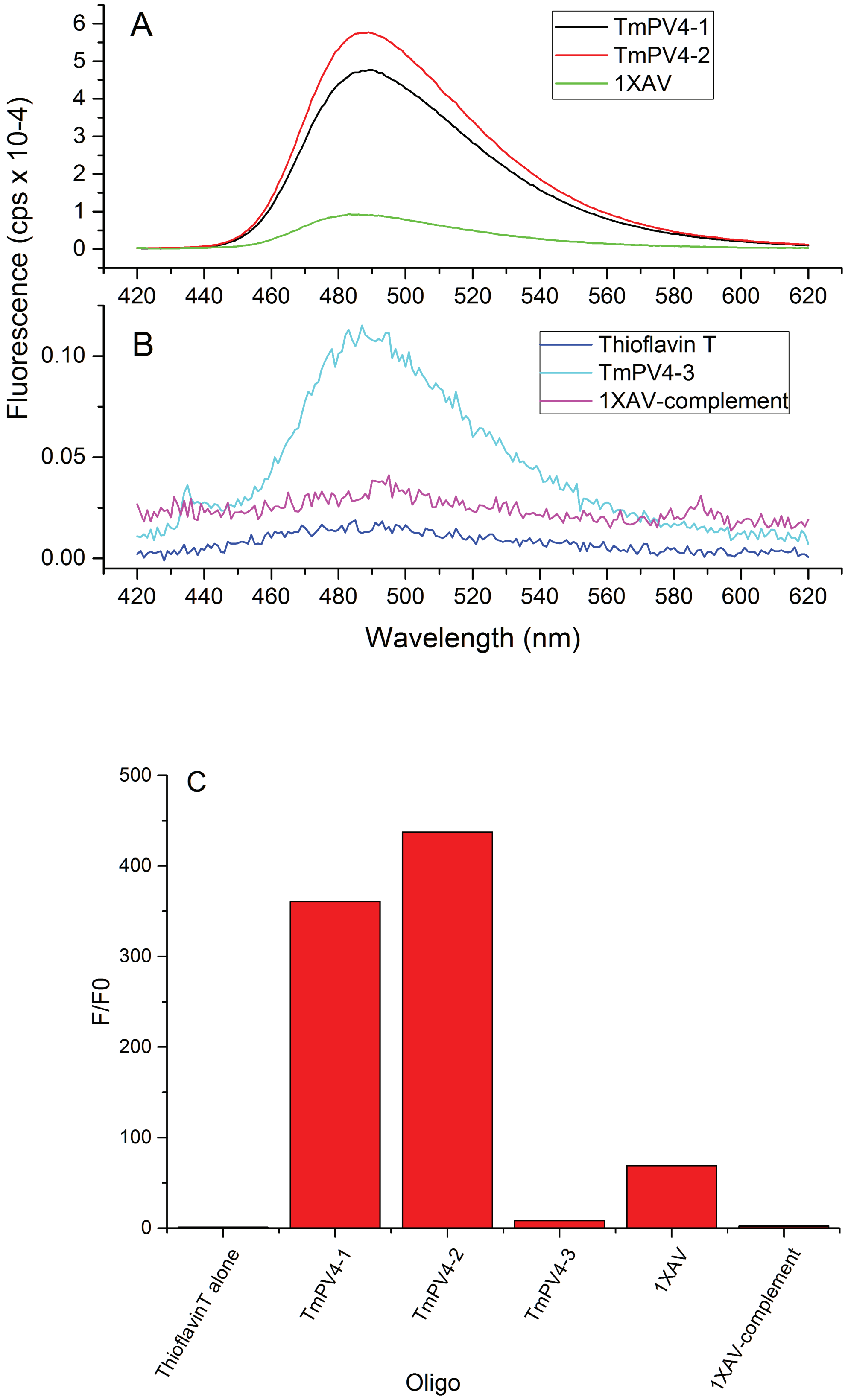

S2 Fig. Thioflavin T fluorescence intensity given in counts/s (cps) in the absence of DNA and in the presence of TmPV4 oligonucleotides and controls 1XAV and 1XAV-complement. Panel A depicts the thioflavin T emission spectra of TmPV4-1, TmPV4-2, and 1XAV. Panel B shows the weak emission of thioflavin T in the absence of DNA as well as in the presence of TmPV4-3 and 1XAV-complement. Panel C shows the fold enhancement F/F_0 of thioflavin T fluorescence in the presence of DNA (F) relative to that of the DNA-free emission intensity (F_0). Conditions: 0.5 µM thioflavin T, 1 µM DNA strand, 10 mM tBAP, 1 mM EDTA, 50 mM KCl, pH 7.0, 25 °C.
